# Using a multilocus phylogeny to test morphology-based classifications of *Polystichum* (Dryopteridaceae), one of the largest fern genera

**DOI:** 10.1186/s12862-016-0626-z

**Published:** 2016-02-29

**Authors:** Timothée Le Péchon, Hai He, Liang Zhang, Xin-Mao Zhou, Xin-Fen Gao, Li-Bing Zhang

**Affiliations:** Chengdu Institute of Biology, Chinese Academy of Sciences, P.O. Box 416, Chengdu, Sichuan 610041 China; School of Life Sciences, University of KwaZulu-Natal, Private Bag X01 Scottsville, Pietermaritzburg, 3209 South Africa; School of Life Sciences, Chongqing Normal University, Shapingba, Chongqing, 400047 China; School of Life Sciences, Sichuan University, Chengdu, Sichuan 610064 China; Missouri Botanical Garden, P.O. Box 299, St. Louis, MO 63166-0299 USA

**Keywords:** *Cyrtomium*, Dryopteridaceae, Fern phylogeny, HYMASO superclade, HYSUFI clade, MCSCHMANS clade, *Phanerophlebia*, *Polystichum*

## Abstract

**Background:**

*Polystichum* (Dryopteridaceae) is probably the third largest fern genus in the world and contains ca. 500 species. Species of *Polystichum* occur on all continents except Antarctica, but its highest diversity is found in East Asia, especially Southwest China and adjacent regions. Previous studies typically had sparse taxon sampling and used limited DNA sequence data. Consequently, the majority of morphological hypotheses/classifications have never been tested using molecular data.

**Results:**

In this study, DNA sequences of five plastid loci of 177 accessions representing ca. 140 species of *Polystichum* and 13 species of the closely related genera were used to infer a phylogeny using maximum likelihood, Bayesian inference, and maximum parsimony. Our analyses show that (1) *Polystichum* is monophyletic, this being supported by not only molecular data but also morphological features and distribution information; (2) *Polystichum* is resolved into two strongly supported monophyletic clades, corresponding to the two subgenera, *P.* subg. *Polystichum* and *P.* subg. *Haplopolystichum*; (3) Accessions of *P.* subg. *Polystichum* are resolved into three major clades: clade K (*P.* sect. *Xiphophyllum*), clade L (*P.* sect. *Polystichum*), and the HYMASO superclade dominated by accessions of *P.* sect. *Hypopeltis*, *P.* sect. *Macropolystichum*, and *P.* sect. *Sorolepidium*, while those of *P.* subg. *Haplopolystichum* are resolved into eight major clades; and (4) The monophyly of the *Afra* clade (weakly supported), the Australasian clade (weakly supported), and the North American clade (strongly supported) is confirmed.

**Conclusions:**

Of the 23 sections of *Polystichum* recognized in a recent classification of the genus, four (*P.* sect. *Hypopeltis*, *P.* sect. *Neopolystichum*, *P.* sect. *Sorolepidium*, *P.* sect. *Sphaenopolystichum*) are resolved as non-monophyletic, 16 are recovered as monophyletic, and three are monospecific. Of the 16 monophyletic sections, two (*P.* sect. *Adenolepia*, *P.* sect. *Cyrtogonellum*) are weakly supported and 14 are strongly supported as monophyletic. The relationships of 11 sections (five in *P.* subg. *Haplopolystichum*; six in *P.* subg. *Polystichum*) are well resolved.

**Electronic supplementary material:**

The online version of this article (doi:10.1186/s12862-016-0626-z) contains supplementary material, which is available to authorized users.

## Background

As one of the most species-rich fern genera, *Polystichum* Roth (Dryopteridaceae) is almost a cosmopolitan genus naturally distributed on every continent except Antarctica. Estimates of the number of species in the genus worldwide have ranged from at least 200 [1], to slightly more than 225 [2], ca. 300 [3, 4] to ca. 380 [5]. Our recent explorations in species-rich areas such as southern China and northern and central Vietnam have demonstrated that there are actually far more species in the genus than had been thought. Besides, the application of molecular phylogenetics in the study of *Polystichum* has revealed a number of cryptic or semi-cryptic species of *Polystichum* previously unknown to science (e.g., [6–19]). Consequently, in its most recent circumscription (i.e., *sensu* [18, 20]), *Polystichum* constitutes likely the third largest fern genus in the world with ca. 500 species [18], just smaller than *Asplenium* L. (ca. 660 spp. [21]; Aspleniaceae) and *Elaphoglossum* Schott ex J. Smith (ca. 600 spp. [22]; Dryopteridaceae). Species of *Polystichum* commonly occur in temperate and subtropical regions, in lowlands and montane to alpine areas, and are most diverse in the Northern Hemisphere, especially in southwestern and southern China, the Himalaya (ca. 50 spp., [2]), Japan (32 spp., [23]), and Vietnam (ca. 40 spp.). The Old World taxa represent ca. 80 % of the species diversity in the genus. A rich diversity of *Polystichum* is also found in the mountains of tropical Americas (e.g., Central America: 18 spp.; Bolivia: 21 spp., [24]; Costa Rica: 12 spp., [25]; Cuba: ca. 17 spp., C. Sánchez, pers. comm.; Mexico: 17 spp., [26]; West Indies: 31 spp., [27]). About 15 species of *Polystichum* are distributed in North America and north of Mexico [28], 16 in mainland Africa [29], eight in Madagascar and the Mascarene Islands [30], three in Macronesia [31], four in Europe [32], 12 in Australasia [33, 34] and a few in New Guinea and the Pacific islands.

A morphology-based infrageneric classification of a group is basically phylogenetic hypotheses based on morphology. An infrageneric treatment of a genus is important for floristic and monographic studies and this is particularly true for a large genus like *Polystichum* [18]. Although infrageneric classifications of *Polystichum* go back at least to Keyserling [35] who established *P.* sect. *Parapolystichum* Keyserling (= *Parapolystichum* (Keyserl.) Ching), the first relatively comprehensive attempt at subdividing the genus in a natural way was conducted by Tagawa [36]. Based on morphological characters such as pinnation, the aspect of scales and sori, Tagawa [36] divided the species of Korea, Japan, and Taiwan into eight sections: *P.* sect. *Achroloma* Tagawa, *P.* sect. *Crucifilix* Tagawa, *P.* sect. *Cyrtomiopsis* Tagawa, “*P.* sect. *Eupolystichum*” Diels (=*P.* sect. *Polystichum*), *P.* sect. *Haplopolystichum*, *P.* sect. *Mastigopteris* Tagawa, *P.* sect. *Metapolystichum* Tagawa, and *P.* sect. *Sorolepidium* (Christ) Tagawa (Table 1).

**Table 1:** Infrageneric classifications of *Polystichum*

Tagawa [36]	Daigobo [37]	Fraser-Jenkins [2, 38]	Kung et al. [4]	Zhang and Barrington [18]
				***P.*** **subg.** ***Polystichum***
*P.* sect. *Achroloma*	*P.* sect. *Achroloma*	-	-	*P.* sect. *Achroloma* (2/2)
*P.* sect. *Polystichum*	*P.* sect. *Polystichum*	*P.* sect. *Polystichum*	*P.* sect. *Polystichum*	*P.* sect. *Polystichum* (9/30)
*P.* sect. *Sorolepidium*	*P.* sect. *Sorolepidium*	*P.* sect. *Sorolepidium*	-	*P.* sect. *Sorolepidium* (7/16)
*P.* sect. *Macropolystichum*	*P.* sect. *Macropolystichum*	*P.* sect. *Macropolystichum*	*P.* sect. *Macropolystichum*	*P.* sect. *Macropolystichum* (8/17)
*P.* sect. *Micropolystichum*	*P.* sect. *Micropolystichum*	*P.* sect. *Micropolystichum*	*P.* sect. *Micropolystichum*	*P.* sect. *Micropolystichum* (2/6)
-	*P.* sect. *Hypopeltis*	*P.* sect. *Hypopeltis*	*P.* sect. *Hypopeltis*	*P.* sect. *Hypopeltis* (46/70)
-	*P.* sect. *Stenopolystichum*	-	*P.* sect. *Stenopolystichum*	*P.* sect. *Stenopolystichum* (2/3)
-	*P.* sect. *Xiphopolystichum*	-	*P.* sect. *Xiphopolystichum*	*P.* sect. *Xiphopolystichum* (14/34)
-	-	-	*P.* sect. *Neopolystichum*	*P.* sect. *Neopolystichum* (2/4)
-	-	-	-	*P.* sect. *Fimbriata* (1/1)
-	-	-	-	*P.* sect. *Hecatoptera* (1/1)
-	-	-	-	*P.* sect. *Crinigera* (1/1)
-	-	-	-	*P.* sect. *Subfimbriata* (1/1)
-	-	-	-	*P.* sect. *Chingiarum* (1/1)
				***P.*** **subg.** ***Haplopolystichum***
*P.* sect. *Cyrtomiopsis*	*P.* sect. *Cyrtomiopsis*	-	-	*P.* sect. *Cyrtomiopsis* (2/4)
*P.* sect. *Crucifix*	*P.* sect. *Crucifix*	-	*P.* sect. *Crucifix*	*P.* sect. *Crucifix* (3/4)
*P.* sect. *Haplopolystichum*	*P.* sect. *Haplopolystichum*	-	*P.* sect. *Haplopolystichum*	*P.* sect. *Haplopolystichum* (6/54)
*P.* sect. *Mastigopteris*	*P.* sect. *Mastigopteris*	-	*P.* sect. *Mastigopteris*	*P.* sect. *Mastigopteris* (1/2)
*P.* sect. *Metapolystichum*	*P.* sect. *Metapolystichum*	*P.* sect. *Metapolystichum*	*P.* sect. *Metapolystichum*	
-	*P.* sect. *Adenolepia*	-	-	*P.* sect. *Adenolepia* (4/6)
-	*P.* sect. *Lasiopolystichum*	-	*P.* sect. *Lasiopolystichum*	-
-	*P.* sect. *Prionolepia*	-	-	-
-	-	*P.* sect. *Duropolystichum*	*P.* sect. *Scleropolystichum*	-
-	*P.* sect. *Sphaenopolystichum*	-	*P.* sect. *Sphaenopolystichum*	*P.* sect. *Sphaenopolystichum* (5/12)
-	-	-	-	*P.* sect. *Basigemmifera* (3/5)
-	-	-	-	*P.* sect. *Cyrtogonellum* (3/5)
-	-	-	-	*P.* sect. *Platylepia* (3/4)

Numbers in the brackets in the last column indicate numbers of species included in our study and the total numbers of species known in the sections

In the study of the species of *Polystichum* of Japan, Ryukyu, and Taiwan, Daigobo [37] recognized Tagawa’s [36] eight sections and proposed an additional eight ones mainly based on the morphology of microscales on abaxial leaf surfaces: *P.* sect. *Adenolepia* Daigobo, *P.* sect. *Lasiopolystichum* Daigobo, *P.* sect. *Macropolystichum* Daigobo, *P.* sect. *Micropolystichum* Daigobo, *P.* sect. *Prionolepia* Daigobo, *P.* sect. *Scleropolystichum* Daigobo, *P.* sect. *Stenopolystichum* Daigobo, and *P.* sect. *Xiphopolystichum* Daigobo (Table 1). Notably, *P.* sect. *Scleropolystichum* is a homotypic synonym of *P.* sect. *Hypopeltis* with *P. aculeatum* as its type [18].

Fraser-Jerkins [2, 38] classified the 45 species of the Indian Subcontinent into seven sections: *P.* sect. *Duropolystichum* Fraser-Jenkins, *P.* sect. *Hypopeltis* (Michx.) T.Moore, *P.* sect. *Macropolystichum*, *P.* sect. *Metapolystichum*, *P.* sect. *Micropolystichum*, *P.* sect. *Polystichum*, and *P.* sect. *Sorolepidium* (Table 1).

In revising the African species of *Polystichum,* Roux [39] classified the 24 species he recognized into nine sections including *P.* sect. *Lasiopolystichum*, *P.* sect. *Metapolystichum*, *P.* sect. *Xiphopolystichum*, and other six sections (*nom. nud.*) he proposed in his Ph.D. dissertation. Later when he published his work [29] he did not describe these six sections officially and recognized only 16 species and one hybrid for Africa. An extensive study of subdividing *Polystichum* was conducted by Kung et al. [4] where the then recognized 168 species of *Polystichum* in China were accommodated in 13 sections. Four of Tagawa’s [36] eight sections and six of Daigobo’s [37] 16 sections were adopted, albeit often with dramatically different circumscriptions. Two additional sections were introduced: *P.* sect. *Neopolystichum* Ching ex Li Bing Zhang & H.S.Kung and *P.* sect. *Sphaenopolystichum* Ching ex W.M.Zhu & Z.R.He (Table 1).

The most recent and comprehensive subdivision of *Polystichum* was performed by Zhang and Barrington [18] who arranged the 208 species recognized for *Flora of China* in two subgenera: *P.* subg. *Polystichum* and *P.* subg. *Haplopolystichum* (Tagawa) Li Bing Zhang, and the former further into 14 sections while the latter into nine sections (Table 1). Nine of the 23 sections were newly proposed and most of the existing sections were circumscribed differently in comparison with their earlier delimitations by Tagawa [36], Daigobo [37], Fraser-Jerkins [2, 38] and Kung et al. [4].

In the era of molecular phylogenetics, although substantial progress in understanding the phylogeny of *Polystichum* has been achieved using plastid *rbcL, rps4-trnS,* and *trnL-F* data [1, 11, 13, 15, 40–45], the relationships among sections, species, and previously recognized genera, *Cyrtogonellum* and *Cyrtomidictyum*, as well as *Cyrtomium* subser. *Balansana*, have not yet been resolved and the majority of the Asian species not included in any molecular analyses. So far no monophyletic supraspecific taxa except *Cyrtomidictyum* (= *P.* sect. *Cyrtomiopsis* Tagawa) have been recovered using molecular data. Almost all morphological hypotheses about the relationships within *Polystichum*, especially in terms of subdivisions of the genus, have largely remained speculative.

The objectives of this study included: (1) to test the monophyly of *Polystichum* using the largest taxon and character sampling so far; (2) to resolve the major relationships within *Polystichum* worldwide with focus on the Old World taxa which represent ca. 80 % of the species diversity in the genus; (3) to evaluate the monophyly of the supraspecific taxa recognized in the most recent classification and to test other previous morphology-based hypotheses of relationships within *Polystichum*.

## Results

**Fig. 1:**
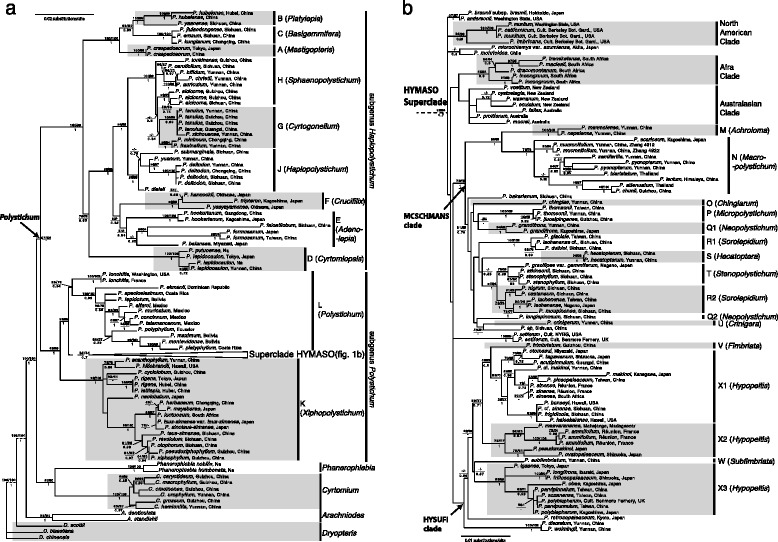
Simultaneous-analysis maximum likelihood tree of *Polystichum* based on five chloroplast markers (*psbA-trnH* intergenic spacer, *rbcL* gene, *rps4-trnS* intergenic spacer*, trnL* intron, and *trnL-F* intergenic spacer) of 177 accessions representing ca. 140 species of *Polystichum* and 13 species of the closely related four genera as outgroups. Maximum parsimony jackknife (MPJK) values/maximum likelihood bootstrap (MLBS) values/Bayesian inference posterior probability (BIPP) values are along the branches. Dashed branches indicate the disproportional branch lengths

This study generated 334 new sequences (Additional file 1). The dataset characteristics and tree statistics for the analyses are presented in Table 2. Comparisons of tree topologies from MPJK analyses of the individual markers did not identify any well-supported conflicts (MPJK ≥ 70 %; [46–48]). Thus, the five datasets were concatenated. The topology of the ML tree based on the concatenated dataset (Fig. 1) is mostly identical to those based on each individual marker, but with generally increased support values.

**Table 2:** Data matrices and best-fitting models for separate (*rbcL*, *psbA-trnH*, *rps4-trnS*, *trnL, trnL-F*, and *trnL* and *trnL-F*) plastid datasets in this study

	*psbA-trnH*	*rbcL*	*trnL-trnL-F*	*trnS-rps4*	Combined
Number of accessions	126	143	141	121	177
Total aligned characters	475	1227	1041	478	3218
% missing data	17.6 %	1.8 %	31.1 %	19.6 %	35,6 %
Number of new sequences	90	65	94	85	334
# PI chars.	76	198	299	178	743
AIC-criterium model	GTR+G	GTR+I+G	GTR+I+G	GTR+I+G	GTR+I+G

*PI* parsimony-informative

*Polystichum* is weakly supported (MLBS: 57 %; MPJK: 58 %; BIPP: < 0.5) as monophyletic and sister to a clade containing *Cyrtomium* and *Phanerophlebia* (Fig. 1a). Within *Polystichum*, the two subgenera, *P.* subg. *Polystichum* (MLBS: 99 %; MPJK: 99 %; BIPP: 1.00) and *P.* subg. *Haplopolystichum* (MLBS: 100 %; MPJK: 99 %; BIPP: 1.00), are strongly supported as monophyletic. Within *P.* subg. *Haplopolystichum*, the 43 accessions of the 34 species are resolved into seven well-supported clades (Fig. 1a: clades A-D, F, G+H, J; MLBS ≥ 96 %; MPJK: ≥ 98 %; BIPP: 1.00) and one weakly supported clade (Fig. 1a: clade E; MLBS: 51 %; MP: unresolved; BIPP: 0.94). Within *P.* subg. *Polystichum*, the 121 accessions of the 106 species are resolved into three well-supported clades: clade K (MLBS: 98 %; MPJK: 100 %; BIPP: 1.00), clade L (MLBS: 85 %; MPJK: 78 %; BIPP: 0.64), and the HYMASO superclade (*Hypopeltis-Macropolystichum-Sorolepidium* dominant superclade; MLBS: 98 %; MPJK: 84 %; BIPP: 0.7), and the HYMASO superclade further into a number of clades including the MCSCHMANS (*Macropolystichum-Chingiarum-Sorolepidium-Crinigera-Hecatoptera-Micropolystichum-Achroloma-Neopolystichum-Stenopolystichum*) clade, the HYSUFI (*Hypopeltis-Subfimbriata-Fimbriata*) clade, the *Afra* clade, the Australasian clade, and the North American clade (Fig. 1b). The HYSUFI clade is weakly supported as monophyletic and comprises clades V, W, and X1–X3, *P. discretum* (Don) J.Sm., *P. retrosopaleaceum* (Kodama) Tagawa, and *P. weimingii* Li Bing Zhang & H.He, while the MCSCHMANS clade is strongly supported as monophyletic (Fig. 1b; MLBS: 78 %; MPJK: 78 %; BIPP: 1.0) and contains clades M–U, *P. bakerianum* (Atkins.) Diels, and an undescribed species from Sichuan, China.

## Discussion

### Monophyly of *Polystichum* and its relationships with *Cyrtomium* and *Phanerophlebia*

While the monophyly of polystichoid ferns (i.e., *Cyrtomium*, *Phanerophlebia* and *Polystichum*) has been highly supported in previous studies [1, 44], the relationships among these three genera remain ambiguous. With the largest sampling so far (about three times as large as the previous largest worldwide sampling by Driscoll and Barrington [44]), our study resolved *Polystichum* as monophyletic but only with weak support (MLBS: 57 %; MPJK: 58 %; BIPP: < 0.50). Although earlier studies [1, 49] using a limited molecular sampling (only *rbcL* sequences) found *Polystichum* (*sensu* [18, 20]) as paraphyletic in relation to *Cyrtomium* (weak support), the monophyly of *Polystichum* is further supported by several morphological features (i.e., lamina 1–3-pinnate, apex pinnatifid, without a clear apical pinna; venation mostly free, rarely anastomosing to form 1 or 2 rows of areoles). Our result is also consistent with some more recent studies based on multi-locus datasets [42, 44, 50].

The sister relationship between *Cyrtomium* and *Phanerophlebia* is highly supported by our phylogenetic reconstructions (MLBS: 86 %; MPJK: 90 %; BIPP: 1.00, Fig. 1a). As early as 1988 Yatskievych et al. already found that *Cyrtomium* and *Phanerophlebia* are convergent descendants from different progenitor groups based on chloroplast restriction site data [51]. However, a closer relationship of *Polystichum* with *Cyrtomium* than with *Phanerophlebia* was found by Li et al. [42] based on plastid *trnL-F* and *rps4-trnS* data and by Mc Keown et al. [50]. Our data do not support this resolution (Fig. 1).

Generally, the relationships among polystichoid ferns obtained here are in agreement with those found in most of the earlier phylogenetic studies but more studies are needed to fully resolve the relationships among these three genera.

### Relationships within *Polystichum*

Within *Polystichum*, 164 accessions are resolved into two monophyletic clades corresponding to *P.* subg. *Polystichum* and *P.* subg. *Haplopolystichum* (Tagawa) Li Bing Zhang defined by [18], both with strong support. The sister relationship between these two subgenera agrees with the morphology and the findings with molecular data by Driscoll and Barrington [44] and Li et al. [42], but contrasts those by Little and Barrington [1] and Lu et al. [49] based on *rbcL* data alone which resolved *P.* subg. *Polystichum* as sister to *Cyrtomium*, and them together as sister to *P.* subg. *Haplopolystichum* (also see above). The bulbil-bearing species are resolved in five clades (A, C, D, N, T), suggesting that bulbils evolved at least five times in *Polystichum*, twice in *P.* subg. *Polystichum* and three times in *P.* subg. *Haplopolystichum*.

I ***Polystichum*** subg. ***Haplopolystichum*** (Tagawa) Li Bing Zhang (Fig. 1a): Nine sections are recognized by Zhang and Barrington [18] in this subgenus. The monophyly of all but two sections is recovered and six sections are well supported as monophyletic. The relationships among all but four sections in the subgenus are well resolved.
  1. *Polystichum* sect. *Mastigopteris* Tagawa (Fig. 1a: clade A). – Morphologically, this section is characterized by having entire indusia [18] and it contains only two species following Zhang and Barrington [18], a delimitation different from that of Kung et al. [4] who included *P. erosum* Ching & Shing in this section as well. Two accessions representing only the type of the section, *P. craspedosorum* (Maxim.) Diels, were included in our study. Our results show that this section is strongly (MLBS: 100 %; MPJK: 98 %; BIPP: 1.00) supported as sister to a clade containing *P.* sect. *Basigemmifera* and *P.* sect. *Platylepia*, and these three sections together are sister to the rest of the subgenus. Our study also shows that *P.* sect. *Mastigopteris sensu* Kung et al. [4] is paraphyletic in relation to portion of *P.* sect. *Basigemmifera* and *P.* sect. *Platylepia* (Fig. 1a: clades B and C).
  2. *Polystichum* sect. *Platylepia* Li Bing Zhang (Fig. 1a: clade B). – This section, characterized by having ovate to broadly lanceolate rachis scales and once-pinnate lamina [18], contains 3–4 species occurring in Southwest to central China. Three accessions representing three species of this section were included in our study. Our data resolved this section as monophyletic (MLBS: 100 %; MPJK: 99 %; BIPP: 1.00) and sister to *P.* sect. *Basigemmifera*. This resolution is not in conflict with what we found earlier based on plastid *trnL-F* data alone [10], which placed *P.* sect. *Platylepia* (represented by *P. yaanense* Liang Zhang & Li Bing Zhang), *P.* sect. *Basigemmifera*, and *P.* sect. *Mastigopteris* in an unresolved trichotomy.
  3. *Polystichum* sect. *Basigemmifera* (W.M.Chu & Z.R.He) Li Bing Zhang (Fig. 1a: clade C). – This section has been accommodated in *P.* sect. *Micropolystichum* by Kung et al. [4] as *P.* ser. *Basigemmifera* W.M.Chu & Z.R.He based on the small size of plants and pinna morphology of the members. However, the type of *P.* sect. *Micropolystichum*, the tetraploid sexual *P. thomsonii* (Hook.f.) Bedd., and its relatives lack bulbils on the rachis and are members of *P.* subg. *Polystichum* (see below), while members of *P.* sect. *Basigemmifera* have bulbils on the rachis and are members of *P.* subg. *Haplopolystichum*. This section contains about five species, four of which are endemic to Southwest to central China [18]. We included three species in this study including the sexual tetraploid *P. erosum* Ching & K.S.Shing. Our data confirmed the monophyly of this section (MLBS: 100 %; MPJK: 100 %; BIPP: 1.00), consistent with our earlier findings based on more species and accessions sampled [10, 15]. Our data resolved this section as sister to *P.* sect. *Platylepia* (Fig. 1a: clade B).
  4. *Polystichum* sect. *Cyrtomiopsis* Tagawa (Fig. 1a: clade D). – This section contains about four species and is characterized by prolonged rachis apex with bulbils and broad-type microscales [18]. This section was often recognized as a genus, i.e., *Cyrtomidictyum* Ching (e.g., [52, 53]). Our data resolved it as part of *P.* subg. *Haplopolystichum*, a result consistent with those in earlier studies [10, 15, 42, 44, 49, 53]. Our study further resolved this section as monophyletic (MLBS: 100 %; MPJK: 100 %; BIPP: 1.00) and sister (MLBS: 78 %; MPJK: 76 %; BIPP: 0.85) to a clade containing *P.* sect. *Adenolepia*, *P.* sect. *Crucifilix*, *P.* sect. *Cyrtogonellum*, *P.* sect. *Haplopolystichum*, and *P.* sect. *Sphaenopolystichum* (all sections of the subgenus except *P.* sect. *Basigemmifera*, *P.* sect. *Mastigopteris*, and *P.* sect. *Platylepia*). This resolution is consistent with that found by Driscoll and Barrington ([44]; maximum parsimony bootstrap: 100 %; 7 species of the subgenus sampled) and that by Lu et al. ([49]; maximum parsimony bootstrap: 67 %; 12 species of the subgenus sampled but *P.* sect. *Mastigopteris* was not sampled), but inconsistent with that found by Li et al. [42] who resolved *P.* sect. *Cyrtomiopsis* (one species sampled) as sister to the rest of *P.* subg. *Haplopolystichum* (maximum parsimony bootstrap: 70 %) based on plastid *trnL-F* and *rps4-trnS* data. Occasionally, veinlets in species of this section can be anastomosing.
  5. *Polystichum* sect. *Adenolepia* Daigobo (Fig. 1a: clade E). – This section in its new circumscription [18] contains about six species including four assigned to *Cyrtomium* in early classifications (e.g., [4]). We sampled four species in our study. Our analyses recovered the monophyly of the section but only with weak support in ML and MP analyses (MLBS: < 50 %; MPJK: < 50 %) but moderately support in BI analysis (BIPP: 0.94). Interestingly, the two former members of *Cyrtomium*, which have anastomosing venation [*P. balansae* Christ, *P. hookerianum* (C.Presl) C.Chr.], are paraphyletic in relation to two species with free venation, suggesting that the anastomosing venation in the section evolved at least twice or evolved once but reversed to free venation from anastomosing venation in the *P. falcatilobum* + *P. formosanum* clade. *Polystichum* sect. *Adenolepia sensu* Daigobo [37], which included *P. obliquum* (Don) T.Moore, a member of *P.* sect. *Haplopolystichum*, is apparently polyphyletic.
  6. *Polystichum* sect. *Crucifilix* Tagawa (Fig. 1a: clade F). – This section contains only four species, three of which were included in our study. Our data confirmed the monophyly of this section (MLBS: 98 %; MPJK: 99 %; BIPP: 1.00) and resolved it as sister to *P.* sect. *Adenolepia*, but this resolution is only weakly supported (MLBS: 53 %; MP: unresolved; BIPP: 0.82). The Japanese endemic *P. yaeyamense* (Makino) Makino is surprisingly resolved as a member of *P.* sect. *Crucifilix.* A close examination of herbarium material of *P. yaeyamense* shows that some individuals of this species have and some do not have bipinnate lamina base, similar to *P. normale* Ching ex P. S. Wang & Li Bing Zhang [54], another member of this section [18].
  7. *Polystichum* sect. *Cyrtogonellum* (Ching) Li Bing Zhang (Fig. 1a: clade G). – This section was often treated as a genus (e.g., [52]) but was recently considered as a section of *Polystichum* by Zhang and Barrington [18]. Liu et al. [53] recovered the monophyly of this section with high MLBS support (95 %). Our earlier *trnL-F* data alone resolved this section as paraphyletic in relation to *P.* subg. *Haplopolystichum* [9]. Our current study recovered the monophyly of the section but with low statistical support in ML and BI analyses (MLBS < 50 %; BIPP: 0.84). Monophyly of *P.* sect. *Cyrtogonellum* is supported by morphology: species of this section have one row of sori on each side of midrib, pinnae symmetrical or nearly symmetrical at the base except *P. minimum* (Y.T.Hsieh) Li Bing Zhang. One species (*P. fraxinellum* (Christ) Diels) and one hybrid (*P.* ×*rupestris* P.S. Wang & Li Bing Zhang) included in this section have anastomosing venation [18]. This, together with anastomosing venation in *P.* sect. *Adenolepia* (see above) and sometimes in *P.* sect. *Cyrtomiopsis*, reinforces that anastomosing venation in *Polystichum* evolved multiple times independently.
  8. *Polystichum* sect. *Sphaenopolystichum* Ching ex W.M.Chu & Z.R.He (Fig. 1a: “clade” H). – This section is the only one in the subgenus with pinnae finely dissected [4, 18] and contains about 15 species. Lu et al. [49] recovered the monophyly of the section (MPBS: 98 %) but only two species were sampled. Liu et al. [53] sampled three species and recovered the monophyly of *P.* sect. *Sphaenopolystichum* but without any statistical support. Our data of eight accessions representing ca. eight species failed to recover the monophyly of the section in all three analyses; instead the eight accessions were resolved into three subclades: the *P. alcicorne* subclade containing ca. three species, the *P. tonkinense* subclade containing one species, and the *P. auriculum* subclade containing *P. auriculum* Ching, *P. bifidum* Ching, *P. caruifolium* (Baker) Diels, and *P. christii* Ching (Fig. 1a: clade H). In BI analysis, the *P. alcicorne* subclade is resolved as sister to *P.* sect. *Cyrtogonellum* with BIPP = 0.60. *P.* sect. *Sphaenopolystichum* together with *P.* sect. *Cyrtogonellum* is strongly supported as monophyletic (MLBS: 100 %; MPJK: 99 %; BIPP: 1.00). One member of the section, *P. wattii* (Bedd.) C.Chr., has never been included in any molecular studies and might not belong to this section.
  9. *Polystichum* sect. *Haplopolystichum* Tagawa (Fig. 1a: clade J). – In its recent circumscription (i.e., *sensu* Zhang and Barrington [18]), *P.* sect. *Haplopolystichum*, is different from its original delimitation by Tagawa [36]. The latter contained also *P.* sect. *Adenolepia*, *P.* sect. *Hecatoptera*, and *P.* sect. *Stenopolystichum* in our definition [18]. Our results show that *P.* sect. *Haplopolystichum sensu* Tagawa [36] is highly polyphyletic and taxa included in its original definition are resolved in two subgenera (see above and our Fig. 1a, b). This section in our definition [18] is estimated to contain about 200 species [18] and almost all species recently described from southern China and Vietnam belong to this section [6–11, 15, 16, 19, 55]. We included seven accessions representing four species. An ongoing project focusing on this section will include many more species. Our current study shows that this section is strongly supported as monophyletic (MLBS: 96 %; MPJK: 98 %; BIPP: 1.00) and is resolved as sister (MLBS: 94 %; MPJK: 99 %; BIPP: 1.00) to a clade containing *P.* sect. *Cyrtogonellum* and *P.* sect. *Sphaenopolystichum*.
II ***Polystichum*** subg. ***Polystichum*:** All accessions of this subgenus are resolved into three major clades: clade K (*P.* sect. *Xiphophyllum*; well supported), clade L (*P.* sect. *Polystichum*; moderately supported), and the HYMASO superclade (MLBS: 98 %; MPJK: 84 %; BIPP: 0.7). The HYMASO superclade represents ca. 30 % of the species diversity in the genus and is morphologically diverse. It contains 11 of the 23 sections recognized by Zhang and Barrington [18] for the genus in China. Within the HYMASO superclade, the relationships are poorly resolved. Nevertheless, we recovered two major clades: the HYSUFI clade (weakly supported) and the MCSCHMANS clade (moderately supported). The former contains species of *P.* sect. *Hypopeltis*, *P.* sect. *Fimbriata*, and *P.* sect. *Subfimbriata*, while the latter contains species of nine sections recognized by Zhang and Barrington [18]. The bulbil-bearing species in the subgenus only appear in the MCSCHMANS clade.
  10 *Polystichum* sect. *Xiphopolystichum* Daigobo (Fig. 1b: clade K). – *Polystichum* sect. *Xiphopolystichum* is defined as a combination of *P.* sect. *Xiphopolystichum sensu* Kung et al. [4] and *P.* sect. *Duropolystichum* Fraser-Jenk. [18]. Fifteen out of ca. 34 species of the section were sampled in our study (the largest sampling so far). This clade, strongly supported as monophyletic (MLBS: 98 %; MPJK: 100 %; BIPP: 1.00), is resolved as sister to the rest of the subgenus with high support values (MLBS: 99 %; MPJK: 99 %; BIPP: 1.00). *P.* sect. *Xiphopolystichum* is also characterized by several morphological features such as the lamina stiff, leathery or nearly leathery, often shiny adaxially; the pinnae dentate and with hard spinules at apex and often also on margin; and the rachis scales linear and brown to blackish [18]. Our resolution is consistent with that found by Driscoll and Barrington [44] who sampled five species only. Within the section (clade K), *P.* sect. *Xiphopolystichum sensu* Kung et al. [4] strongly supported as monophyletic (MLBS: 99 %; MPJK: 100 %; BIPP: 1.00), while *P.* sect. *Scleropolystichum* (= *P.* sect. *Duropolystichum*) *sensu* Kung et al. [4] is paraphyletic in relation to *P.* sect. *Xiphopolystichum sensu* Kung et al. [4]. Morphologically, *P.* sect. *Duropolystichum* could well be monophyletic based on its open spines on the pinna margins and thicker leaves [4]. More molecular data might recover the monophyly of *P.* sect. *Duropolystichum*.
  11 *Polystichum* sect. *Polystichum* (Fig. 1b: clade L). – Ten accessions representing nine species of this section are resolved as the second earliest divergent lineage in the subgenus (MLBS: 85 %; MPJK: 78 %; BIPP: 0.64), which is consistent with the findings by Driscoll and Barrington [44] who first discovered this clade. Species of this section occur in the circumboreal regions (*P. lonchitis* (L.) Roth, a diploid sexual species) and the New World tropics [18, 44]. McHenry and Barrington [45] further discovered that the exindusiate Andean *Polystichum* was sister to the Mexican *Polystichum* clade and they together were sister to the Mexican *P. speciosissimum* (Kunze) R.M.Tryon & A.F.Tryon. Morphologically, this section is characterized by having lamina papery or leathery, 1-pinnate to bipinnate; pinna or pinnule spinulose or not spinulose on the margins; and sori indusiate or exindusiate [18, 45]. The currently defined *P.* sect. *Polystichum sensu* Driscoll and Barrington [44] and Zhang and Barrington [18] might need further division based on the results of McHenry and Barrington [45].
    *Polystichum* sect. *Polystichum sensu* Fraser-Jenkins [38] also included the tetraploid sexual *P. acutidens* Christ and four diploid sexuals *P. atkinsonii* Bedd., *P. nepalense* (Spr.) C.Chr., *P. obliquum* (D.Don) T.Moore, and *P. stenophyllum* Christ. Our study, together with previous studies (e.g., [9, 10] for the relationships of *P. acutidens* and *P. obliquum*), shows that *P.* sect. *Polystichum sensu* Fraser-Jenkins [38] is apparently polyphyletic as these members are resolved in three independent clades (Fig. 1a and b: clades J, L, T).
  12 *Polystichum* sect. *Achroloma* Tagawa (Fig. 1b: clade M). – When Tagawa [36] established this section, he included only the type *P. nepalense* (Spr.) C.Chr. (a diploid sexual species). Daigobo [37] added *P. falcatipinnum* Hayata (= *P. manmeiense* (Christ) Nakaike, tetraploid sexual) to this section. This delimitation was accepted by Zhang and Barrington [18] but rejected by Kung et al. [4] who included the two species in *P.* sect. *Polystichum*. Fraser-Jenkins [38] placed the latter species in *P.* sect. *Hypopeltis*. Our study is the first to sample both of the species in a molecular analysis. Two species formed a clade with strong support (MLBS: 100 %; MPJK: 100 %; BIPP: 1.00) in our analysis. This section is resolved as sister to the *P.* sect. *Macropolystichum* clade (MLBS: 85 %; MPJK: 85 %; BIPP: 1.0), consistent with the resolution found by Driscoll and Barrington [44]. These two sections share evergreen leaves which are shiny adaxially.
  13 *Polystichum* sect. *Macropolystichum* Daigobo (Fig. 1b: clade N). – As defined by Zhang and Barrington [18], this section contains species with or without proliferous bulbils but all members are of relatively large habit and laminae that are dark green and shiny adaxially. Ten accessions representing about 8 out of ca. 17 species of this section are sampled in our study (the largest sampling so far). Our analyses recovered the monophyly of *P.* sect. *Macropolystichum sensu* Zhang and Barrington [18] with strong support (MLBS: 78 %; MPJK: 73 %; BIPP: 1.00). *Polystichum* sect. *Macropolystichum sensu* Daigobo [37] which includes *P. kiusiuense* Tagawa (= *P. grandifrons* C.Chr.; [56]) is apparently polyphyletic. The type of *P.* sect. *Prionolepis* Daigobo, the tetraploid sexual *P. biaristatum* (Blume) T.Moore, is resolved as a member of *P.* sect. *Macropolystichum sensu* Zhang and Barrington [18] suggesting that *P.* sect. *Prionolepis* is a heterotypic synonym of *P.* sect. *Macropolystichum*. The same species was treated as a member of *P.* sect. *Neopolystichum* by Zhang and Kung [57], but this is not supported by our data. *P. mucronifolium*, resolved as a member of *P.* sect. *Macropolystichum* in our study, was placed in *P.* sect. *Metapolystichum* Tagawa, a heterotypic synonym of *P.* sect. *Hypopeltis* [18], by Fraser-Jenkins [38].
  14 *Polystichum* sect. *Chingiarum* Li Bing Zhang (Fig. 1b: clade O). – This monospecific section contains *P. chingiae* Ching [18] and our study is the first to include it in a molecular analysis. Our study resolved the species in the MCSCHMANS clade, but its relationships are not well resolved. The isolated phylogenetic position is consistent with its special morphology. Morphologically, this species has lamina 1-pinnate, pinnae not cartilaginous at margins, and sori in 2 or 3 rows on each side of midrib and abaxial on veinlets [18]. Such a combination of morphological features is unique within the genus.
  15 *Polystichum* sect. *Micropolystichum* Tagawa (Fig. 1b: clade P). – This section contains only about six montane to alpine species [18]. We included three accessions representing two species. Our study resolved *P.* sect. *Micropolystichum* as a strongly supported clade (MLBS: 100 %; MPJK: 100 %; BIPP: 1.00) which is sister to *P. grandifrons*, but the sister relationship between those lineages is weakly supported statistically (MLBS: < 50 %; MPJK: < 50 %) and morphologically [18].
    Fraser-Jenkins [38] also placed the diploid sexual *Polystichum capillipes* (Baker) Diels and *P. wattii* (Bedd.) C.Chr. in *P.* sect. *Micropolystichum*. Neither of the species were sampled in our current study, but our earlier study [10] resolved the former species in *P.* sect. *Basigemmifera* (Fig. 1a: clade C).
  16 *Polystichum* sect. *Neopolystichum* Ching ex Li Bing Zhang & H.S.Kung (Fig. 1b: clade Q). – When Zhang and Kung [57] described this section, seven species were included. Zhang and Barrington [18] excluded *P. biaristatum* (Blume) T.Moore, *P. mucronifolium* (Blume) C.Presl, and *P. parvifoliolatum* W.M.Chu from the section *sensu* Zhang and Kung [57] but recognition of this section was tentative pending more evidence. We included three accessions representing two species in this study: *P. grandifrons* and *P. longispinosum* Ching ex Li Bing Zhang & H.S.Kung. Our data failed to recover the monophyly of the section although both species are resolved as members of the MCSCHMANS clade (Fig. 1b). Fraser-Jenkins [38] placed *P. grandifrons* C.Chr. in *P.* sect. *Macropolystichum*, which is not supported by our data. Given our limited phylogenetic sampling and the low support values, the taxonomic rearrangements in *P.* sect. *Neopolystichum* need further investigations.
  17 *Polystichum* sect. *Sorolepidium* (Christ) Tagawa (Fig. 1b: clade R). – *Sorolepidium* Christ was often recognized as a genus (e.g., [52, 58]). Liu et al. [59] found it being nested within *Polystichum* based on *rbcL* data. Zhang and Barrington [18] recognized it as a section of *Polystichum* and redefined it as being comparable to *P.* ser. *Moupinensia* H.S.Kung & Li Bing Zhang, only a part of *P.* sect. *Lasiopolystichum sensu* Kung and Zhang [60]. Eight accessions representing ca. seven species are included in our study. The section is resolved as paraphyletic in relation to *P. nudisorum* Ching (a member of *P.* sect. *Hypopeltis*) and *P.* sect. *Stenopolystichum* (see below). Our data suggest that it might be necessary to recognize *P.* sect. *Sorolepidium sensu stricto* (*P. duthiei* (C.Hope) C.Chr. and *P. glaciale* Christ; Fig. 1b: clade R1; MLBS: 100 %; MPJK: 100 %; BIPP: 1.00) and *P.* sect. *Lasiopolystichum* Daigobo (Fig. 1b: clade R2; MLBS: 78 %; MPJK: 77 %; BIPP: 1.0).
    *Polystichum* sect. *Sorolepidium sensu* Fraser-Jenkins [38] also included the diploid sexual *P. bakerianum* (Atk. ex C.B. Clarke) Diels and the tetraploid sexual *P. wilsonii* Christ which were included in *P.* sect. *Hypopeltis sensu* Zhang and Barrington [18]. Our study clearly placed *P. wilsonii* as a member of clade X1 and the phylogenetic position of *P. bakerianum* is not resolved (Fig. 1b).
  18 *Polystichum* sect. *Hecatoptera* (L.L.Xiang) Li Bing Zhang (Fig. 1b: clade S). – This monospecific section contains *P. hecatopterum* Diels only [18], a diploid sexual [61], and our study is the first to include this species in a molecular analysis. We could not amplify its *rbcL* gene. Our data from other four plastid loci show that this species is definitely a member of *P.* subg. *Polystichum* confirming our earlier hypothesis [18], in spite of its striking morphological similarities with members of *P.* subg. *Haplopolystichum* in once-pinnate lamina without bulbils on its rachis [18]. Xiang [62] established *P.* ser. *Hecatoptera* L.L.Xiang based on its long-spinulose pinna margins but placed it in *P.* sect. *Haplopolystichum*, as Tagawa [36] did. Interestingly, Daigobo [37] placed this species in *P.* sect. *Stenophyllum*, which is a section of *P.* subg. *Polystichum* (see our discussion below) although he did not recognize any subgenera in the genus. Our ML and BI analyses resolved *P. hecatopterum* as sister to a clade containing *P.* sect. *Stenopolystichum* and part of *P.* sect. *Sorolepidium* (R2) with weak support.
  19 *Polystichum* sect. *Stenopolystichum* Daigobo (Fig. 1b: clade T). – Tagawa [36] placed the type of the section, *P. stenophyllum* Christ, a diploid sexual species, in *P.* sect. *Haplopolystichum* based on its once-pinnate lamina and terminal sori on veinlets. Four accessions representing ca. three species of this section are included in our study. All species of this section have proliferous bulbils at the apex of lamina [18, 37]. Our study is the first to confirm the monophyly of the section. This section is resolved as monophyletic (MLBS: 75 %; MPJK: 59 %; BIPP: 0.99) and sister to *P.* sect. *Lasiopolystichum sensu* Daigobo [37] with high support values (MLBS: 97 %; MPJK: 94 %; BIPP: 1.00). This sister relationship is unexpected given the huge differences between the two sections. *Polystichum* sect. *Lasiopolystichum* was merged with *P.* sect. *Sorolepidium* by Zhang and Barrington [18].
  20 *Polystichum* sect. *Crinigera* Li Bing Zhang (Fig. 1b: clade U). – This monospecific section contains *P. crinigerum* (C.Chr.) Ching only [18] and our study is the first to include it in a molecular analysis. *P. crinigerum*, together with *P. nepalense* and *P. chingiae* Ching, was included in *P.* sect. *Polystichum* by Kung et al. [4]. Our study shows that *P. crinigerum* is not closely related to either of them suggesting that the similarity among them in once-pinnate lamina and asymmetrical pinna base is not a synapomorphy. However, the relationships of *P. crinigerum* are not well resolved with our data. Our ML and MP analyses resolved it as sister (MLBS: 53 %; MPJK: 51 %) to a species of *P.* sect. *Hypopeltis* and they together are sister (MLBS < 50 %) to *P. longispinosum* Ching ex Li Bing Zhang & H.S.Kung, a species assigned to *P.* sect. *Neopolystichum* [18, 57]. Our BI analysis resolved it as sister to *P. longispinosum* (BIPP: 0.73).
  21 *Polystichum* sect. *Fimbriata* Li Bing Zhang (Fig. 1: clade V). – This monospecific section contains *P. fimbriatum* Christ [18] and our study is the first to include it in a molecular analysis. *Polystichum fimbriatum* is strongly (MLBS: 88 %; MPJK: 82 %) supported as sister to a clade containing portions of *P.* sect. *Hypopeltis* (Fig. 1b: X1) in our sampling (see below). This sister relationship is unexpected given their dissimilarity in morphology of lamina shape and scales [18]. This resolution collapsed in BI analysis which resolved it as part of a trichotomy.
  22 *Polystichum* sect. *Subfimbriata* Li Bing Zhang (Fig. 1b: clade W). – This monospecific section contains *P. subfimbriatum* W.M.Chu & Z.R.He [18] and our study is the first to include it in a molecular analysis. When *P. subfimbriatum* was described, Chu and He [63] compared it with *P. fimbriatum*. Indeed, both species share once-pinnate and leathery lamina, but their scales on rachis and stipes are very different. A close relationship between these two species is not suggested with our analyses which resolved *P. subfimbriatum* as sister to portion of *P.* sect. *Hypopeltis* (Fig. 1b: X3; MLBS: < 50 %; BIPP: 0.57) but with low statistical support.
  23 *Polystichum* sect. *Hypolepis* (Michx.) T.Moore (Fig. 1b: clade X). – Zhang and Barrington [18] re-defined this section and made it the most accommodating section in the genus. They noted that this section in their definition is possibly polyphyletic. We included 55 accessions in our study. Our results show that *P.* sect. *Hypolepis* is indeed polyphyletic. Accessions of this section are resolved in about nine subclades, which partially corresponds to the morphological heterogeneity noticed in this section. Although polyphyletic, the majority of species belonging to *P.* sect. *Hypolepis* are included in the HYSUFI clade which also contains *P. fimbriatum* and *P. subfimbriatum*. Within this clade, three relatively well-supported subclades can be identified: the *P. ovatopaleaceum* subclade (Fig. 1b: subclade X2), the *P. polyblepharum* subclade (Fig. 1b: subclade X3), and the *P. sinensis* subclade (Fig. 1b: subclade X1). The *P. polyblepharum* subclade is strongly supported as monophyletic (MLBS: 99 %; MPJK: 96 %; BIPP: 1.00; subclade X3) and contains species from Japan (e.g., *Polystichum igaense* Tagawa), Taiwan (e.g., *Polystichum sozanense* Ching ex H. S. Kung & Li Bing Zhang) and eastern China (e.g., *Polystichum polyblepharum* (Roemer ex Kunze) C.Presl). The *P. ovatopaleaceum* subclade is also supported as monophyletic (MLBS: 72 %; MPJK: 77 %; BIPP: 1.00; subclade X2) and contains species from East China (e.g., *Polystichum ovatopaleaceum* (Kodama) Sa. Kurata), Japan (e.g., *Polystichum pseudomakinoi* Tagawa), and the Mascarenes (e.g., *Polystichum ammifolium* (Poir.) C.Chr.). The *P. sinensis* subclade (MLBS: 89 %; MPJK: 73 %; BIPP: < 0.5; subclade X1) contains species from Africa, China (especially West China), Hawaii, Japan, and the Mascarenes. Species of the *P. sinensis* subclade is also characterized by their lanceolate rachis scales.
    Uncertainties still remain regarding the phylogenetic positions of several species previously assigned to *P.* sect. *Hypopeltis*, i.e., *P. bakerianum* (Atkins.) Diels, *P. braunii* (Spenn.) Fée, *P. discretum* (Don) J.Sm., *P. microchlamys* (Christ) Kodama, *P. retrosopaleaceum* (Kodama) Tagawa, *P. setiferum* (Forssk.) Moore ex Woynar (type of the section), *P. weimingii* Li Bing Zhang & H.He, members of the Australasian clade [40], and members of the *Afra* clade [44]. But these issues do not affect our overall topology. The monophyly of *P.* sect. *Achroloma*, *P.* sect. *Macropolystichum*, *P.* sect. *Polystichum*, *P.* sect. *Sorolepidium*, *P.* sect. *Stenopolystichum*, and *P.* sect. *Xiphopolystichum* is strongly supported by our analyses (Fig. 1b).
    Notably, the isolated positions of these species is in line with their peculiar morphology. *Polystichum discretum* (diploid) and *P. weimingii* were placed in *P.* ser. *Linearia* H.S.Kung & Li Bing Zhang based on their linear stipe scales by Zhang and Kung [64] and Zhang and Barrington [18], and our current study resolved them as sister to each other with strong support (MLBS: 100 %; MPJK: 100 %; BIPP: 1.00), confirming their close relationships with each other hypothesized by Zhang and He [7]. *Polystichum bakerianum* (diploid) and *P. microchlamys* (diploid or triploid) do not seem to have close relatives judging from their morphologies. A close relative of *P. braunii* (tetraploid), *P. ningshenense* Ching & Y.P.Hsu, as hypothesized by Zhang and Kung [64], is not sampled in our current study. Zhang and Kung [64] established *P.* ser. *Brauniana* H.S.Kung & Li Bing Zhang to accommodate *P. braunii*, *P. ningshenense*, and some species in our subclades X2 and X3 (Fig. 1b). Our current study did not recover the monophyly of *P.* ser. *Brauniana*.
  24 The *Afra,* the North American, and the Australasian lineages (Fig. 1b: *Afra* clade, the North American clade, and the Australasian clade). – In our topology, several African species are grouped together in a clade with moderate support (MLBS: 65 %; MPJK: 54 %; BIPP: 0.90). This group was previously circumscribed and named the *Afra* clade by Driscoll and Barrington [44]. Unfortunately, our data do not well resolve the relationships of this lineage within *Polystichum*. Indeed, in the ML topology, the *Afra* clade is grouped together with a clade containing only species restricted to Australasia but this sister relationship is not supported by our analyses (MLBS: < 50 %; MP: unresolved; BI: unresolved). The Australasian group (MLBS: < 50 %; MP: unresolved; BI: unresolved) previously identified by Perrie et al. [40] and Li et al. [65] is not supported by our expanded dataset. Four accessions representing three species from North America constitute a well-supported group, consistent with the allozymic evidence by Soltis et al. [66] who found close relationships between two species of this clade [*P. imbricans* (D.C. Eaton) D.H. Wagner and *P. munitum* (Kaulf.) C.Presl]. American and African clades may deserve a taxonomic recognition as sections of *P.* subg. *Polystichum*. However, our limited taxonomic sampling clearly needs to be expanded and the relationships better resolved to unambiguously assess the monophyly and determine their relationships within the phylogeny of *Polystichum*.

## Conclusions

Our study based on the largest character sampling and most taxonomically comprehensive sampling so far successfully resolved the 164 accessions representing ca. 140 species of *Polystichum* into two well-supported major clades, corresponding to the two subgenera, *P.* subg. *Polystichum* and *P.* subg. *Haplopolystichum*. Although our study is still preliminary of many results, given that the taxon and character sampling still needs improvements and that some results are poorly supported, our current work is the first toward a new classification based on morphological and molecular evidence in the genus *Polystichum*. Of the 23 sections of *Polystichum* recognized in a recent classification of the genus, except three monospecific sections which are each represented by one accession, four sections (*P.* sect. *Hypopeltis*, *P.* sect. *Neopolystichum*, *P.* sect. *Sorolepidium*, *P.* sect. *Sphaenopolystichum*) are resolved as paraphyletic or polyphyletic, 16 are recovered as monophyletic. Of the 16 monophyletic sections, two (*P.* sect. *Adenolepia*, *P.* sect. *Cyrtogonellum*) are weakly supported and 14 are strongly supported. In addition, our study also recovered the monophyly of the *Afra* clade (moderately supported) and the North American clade (strongly supported). The relationships of 11 sections (5 in *P.* subg. *Haplopolystichum*; 6 in *P.* subg. *Polystichum*) are well resolved (MLBS ≥ 78 %; MPJK ≥ 76 %). However, several phylogenetic uncertainties remain, particularly in *P.* sect *Hypopeltis*. These issues probably linked to introgression and/or fast radiation highlight the fact that more data including nuclear data are needed to obtain a complete picture of the evolutionary relationships in polystichoid ferns and therefore draw a new taxonomic framework for one of the largest genera of ferns, *Polystichum*.

## Methods

### Taxonomic sampling

To test the monophyly of the two subgenera and 23 sections recognized in the most recent classification of *Polystichum* [18], we included 121 accessions representing about 106 species of *P.* subg. *Polystichum* and 43 accessions representing 34 species of *P.* subg. *Haplopolystichum* (see Table 1)*.* Specifically, we sampled at least one species for each of the 23 sections including the monospecific sections *P.* sect. *Chingiarum* Li Bing Zhang, *P.* sect. *Crinigera* Li Bing Zhang, *P.* sect. *Fimbriata* Li Bing Zhang, *P.* sect. *Hecatoptera* (L.L.Xiang) Li Bing Zhang, and *P.* sect. *Subfimbriata* Li Bing Zhang. The bitypic *P.* sect. *Mastigopteris* Tagawa is represented by one species, and all other non-monospecific sections by 2 to 46 species. In detail, *P.* sect. *Achroloma* Tagawa was represented by two species, *P.* sect. *Adenolepia* Daigobo by four species, *P.* sect. *Basigemmifera* (W.M.Chu & Z.R.He) Li Bing Zhang by three species, *P.* sect. *Crucifilix* Tagawa by three species, *P.* sect. *Cyrtomiopsis* Tagawa by two species, *P.* sect. *Cyrtogonellum* (Ching) Li Bing Zhang by three species, *P.* sect. *Haplopolystichum* Tagawa by six species, *P.* sect. *Hypopeltis* (Michx.) T.Moore by 46 species, *P.* sect. *Macropolystichum* Daigobo by eight species, *P.* sect. *Micropolystichum* Tagawa by two species, *P.* sect. *Neopolystichum* Ching ex Li Bing Zhang & H.S.Kung by two species, *P.* sect. *Platylepia* Li Bing Zhang by three species, *P.* sect. *Polystichum* by nine species, *P.* sect. *Sorolepidium* (Christ) Tagawa by seven species, *P.* sect. *Sphaenopolystichum* Ching ex W.M.Chu & Z.R.He by five species, *P.* sect. *Stenopolystichum* Daigobo by two species, and *P.* sect. *Xiphopolystichum* Daigobo by 14 species. To make our study more taxonomically meaningful, we sampled the type species of all supraspecific taxa recognized by Zhang and Barrington [18]. Our overall sampling represents almost all major diversity centers of *Polystichum* except the Indonesian-Papuan region. The detailed sampling sites are listed in the Additional file 1. Field work permissions were not required for all the sampling sites except Réunion for which the permission was issued by the National Park of Réunion.

Given that *Cyrtomium* (*sensu* [18]) and *Phanerophlebia* are both monophyletic [42, 43, 51, 67] and each mainly distributed in only one area (i.e., East Asia and southwestern U.S.A. to Central America, respectively), we included six species of *Cyrtomium* and two of *Phanerophlebia*. Denser sampling of these two genera will be performed in a separate ongoing study (Le Péchon et al., unpubl. data). Based on previous molecular [1, 41, 42, 44, 49, 59, 68–70] and morphological works [71], five species of *Dryopteris* Adans. and two of *Arachniodes* Blume were included as outgroups. In total, 177 accessions representing ca. 153 species in the subfamily Dryopterioideae (*sensu* [71]) were included in this study. Taxa included, their classification, voucher information, and GenBank accession numbers are given in Additional file 1.

### DNA extraction, PCR amplification and sequencing

Genomic DNA was extracted from fresh, silica-gel dried, or herbarium leaf fragments using TIANGEN plant genomic DNA extraction kit (TIANGEN Biotech., Beijing, China) according to the manufacturer’s protocols. We selected five chloroplast regions (the intergenic spacers *psbA-trnH*, *trnS-rps4* and *trnL-trnF*, the *trnL* intron, and the protein-coding gene *rbcL*). The primers used to amplify these regions were based on previous studies or newly designed (Table 3). The PCR protocols followed Zhang et al. [72] and Small et al. [73]. All regions were amplified in 25 μL volumes, with 15.85 μL deionized sterile water, 2.5 μL of 25 mol/L *EasyTaq* Buffer, 1.5 mL of 25 mmol/L MgCl_2_ solution, 2 μL of a 2.5 mmol/L dNTP solution in equimolar ratio, 1 μL of each primer at 10 pmol/μL, 1 unit (0.2 μL) of *EasyTaq* DNA polymerase (TransGen Biotech, Beijing, China), and 1 μL of the template DNA. PCR products were purified and sequenced by Invitrogen (Shanghai, China).

**Table 3:** Selected molecular markers and their primers used in this study

Molecular marker	Primer name	Sequence (5′➔3′)	References
*psbA-trnH*	psbA	GTT ATG CAT GAA CGT AAT GCT C	[85]
	trnH	CGC GCA TGG TGG ATT CAC AAT CC	[86]
*rbcL*	rbcL-1F	ATGTCACCACAAACAGAAACTAAAGC	[87]
	rbcL-595F	AAT TCY CAR CCR TTC ATG CGT	This study
	rbcL-895R	AGC TAA GCT GGT RTT KGC RGT	This study
	rbcL-1375R	TCACAAGCAGCAGCTAGTTCAGGACTC	[88]
*rps4-trnS*	trnS	ATG AAT T(A/G)T TA G TTG TTG AG	[89]
	rps4	TAC CGA GGG TTC GAA TC	[90]
*trnL-trnL-trnF*	fern1	GGCAGCCCCCARATTCAGGGRAACC	[91]
	F	ATTTGAACTGGTGACACGAG	[92]

### Sequence alignment and phylogenetic reconstruction

The resulting sequences were edited and assembled with Sequencher V.4.14 (GeneCodes Corporation, Ann Arbor, Michigan, USA). We manually performed the sequence alignment using Bioedit [74]. Gaps (insertion/deletion events) were considered as missing data. Phylogenetic relationships were reconstructed using maximum parsimony (MP), maximum likelihood (ML), and Bayesian inference (BI). Maximum parsimony jackknife (MPJK) analyses [75] were conducted using PAUP* for each dataset with the removal probability set to approximately 37 %; and “jac” resampling emulated. One thousand replicates were performed, each from a different random addition sequence tree, with 100 TBR searches per replicate and a maximum of 100 trees held per TBR search. A final simultaneous MP analysis [76, 77] was conducted based on the combined dataset including the five molecular markers.

Each DNA region of the concatenated molecular matrix was assigned a separate GTR+I+G substitution model. ML tree searches and 10,000 rapid bootstrapping (MLBS) were conducted using RAxML-HPC and default parameters, followed by a search for the best-scoring tree, in a single run [78, 79].

jModelTest 2 [80] was used to select the best-fit likelihood model for Bayesian analyses. The Akaike information criterion [81] was used to select among models. The models selected were GTR+G (*psb-trnH* spacer), GTR+I+G (*rps4-trnS* spacer*,* the combined region *trnL-trnF* and *rbcL* gene). The selected models (Table 2) were then used for tree searches from the respective data partitions in combination. BI analyses were performed using MrBayes v3 [82]. For each DNA partition, we used the appropriate model selected by jModelTest 2, and each molecular region has independent parameters and the overall rate is allowed to be different across partitions. Four chains (i.e., three heated and one cold) of Metropolis-coupled Markov chain Monte Carlo were performed for 50 million generations, sampling every 1000th generation. After checking the convergence of parameter traces among generations using Tracer [83], we discarded the first 25 % of sampled trees as a “burn-in phase”. The remaining trees were then used to calculate Bayesian inference posterior probability (BIPP).

ML and BI analyses were run on the CIPRES cluster available at http://www.phylo.org/ [84].

## Availability of supporting data

DNA sequence alignments and tree are available in the TreeBase (https://treebase.org/treebase-web/search/study/summary.html?id=18607). Taxa sampled with information related to taxonomy, voucher information, and GenBank accession numbers are available in Additional file 1.

## Additional file

Additional file 1: List of taxa sampled with information related to taxonomy, voucher information, GenBank accession numbers. * denotes when only the intergenic spacer *trnL-trnF* is available. Na: data not available. (DOC 96 kb)

## Abbreviations

BI: bayesian inferences
BIPP: bayesian inference posterior probabilities
HYMASO: clade including the sections *Hypopeltis-Macropolystichum-Sorolepidium*
HYSUFI: clade including the sections *Hypopeltis-Subfimbriata-Fimbriata*
MCSCHMANS: clade including the sections, *Macropolystichum-Chingiarum-Sorolepidium-Crinigera-Hecatoptera-Micropolystichum-Achroloma-Neopolystichum-Stenopolystichum*
ML: maximum likelihood
MLBS: maximum likelihood bootstrap
MP: maximum parsimony
MPJK: maximum parsimony jackkniffe
