# Supplementary material for: Using a multilocus phylogeny to test morphology-based classifications of Polystichum (Dryopteridaceae), one of the largest fern genera
Source: BMC Evol Biol. 2016 Feb 29;16:55. doi: 10.1186/s12862-016-0626-z (PMC4772321; doi:10.1186/s12862-016-0626-z)
Supplement: Additional file 1: — List of taxa sampled with information related to taxonomy, voucher information, GenBank accession numbers. * denotes when only the intergenic spacer trnL-trnF is available. Na: data not available. (DOC 96 kb) [file 12862_2016_626_MOESM1_ESM.doc]

**Additional file**. List of taxa sampled with information related to taxonomy, voucher information, GenBank accession numbers. * denotes when only the intergenic spacer *trnL-trnF* is available. Na: data not available.

***Arachniodes denticulata*** (Sw.) Ching, cult., Kromer 2550 (UC): *rps4-trnS* JN189207, *psbA-trnH* JN189425, *trnL*-*trnL-trnF* JN189102*, *rbcL* JN189533; ***Arachniodes standishii*** (T. Moore) Ohwi, cult., X.C., Zhang 3468 (PE): *rps4-trnS* EF540709*,* *trnL*-*trnL-trnF* EF540700, *rbcL* EF540722, Kagoshima, TNS 762593 (TNS): *psbA-trnH* AB575713.

***Cyrtomium caryotideum*** C. Presl, Guizhou, China, Li Bing Zhang et al. 5847 (CDBI, CTC, MO): *rps4-trnS* KU244913*, psbA-trnH* KU244825, *trnL*-*trnL-trnF* KU245000*, rbcL* KU244741; ***Cyrtomium grossum*** Christ, Guizhou, China, LJM 028 (KUN): *trnL*-*trnL-trnF* AY736341 *rbcL* AY694805; ***Cyrtomium hemionitis*** Christ, Yunnan China, LJM 012 (KUN): *trnL*-*trnL-trnF* AY736338***,*** *rbcL* AY694802; ***Cyrtomium macrophyllum*** (Makino) Tagawa, Guizhou, China, Li Bing Zhang 5771 (CDBI, CTC, MO): *rps4-trnS* KU244912*, psbA-trnH* KU244824, *trnL*-*trnL-trnF* KU244999*, rbcL* KU244740; ***Cyrtomium omeiense*** Ching & Shing, Guizhou, China, Li Bing Zhang 5957 et al. (CDBI, CTC, MO): *rps4-trnS* KU244914*, psbA-trnH* KU244826, *trnL*-*trnL-trnF* KU245001*, rbcL* KU244742; ***Cyrtomium urophyllum*** Ching, Yunnan, China, Lu SG/J28 (Na): *rps4-trnS* DQ202448, *rbcL* AY545492; Sichuan, China, LJM 043 (KUN): *trnL*-*trnL-trnF* AY736333.

***Dryopteris bissetiana*** (Baker) C. Chr., China, Moran (COLO): *rps4-trnS* JN189261*, psbA-trnH* JN189479, *trnL-trnL*-*trnF* AY268796**, rbcL* JN189587; ***Dryopteris chinensis*** Koidz., Muju, South Korea, Zhang 2399 (UC): *rps4-trnS* JN189215*, psbA-trnH* JN189433, *trnL-trnL-trnF* JN189110**,* Anhui, China, Liang Zhang and Zhangming Zhu 1114 (CDBI): *rbcL* JX535859; ***Dryopteris scottii*** (Bedd.) Ching, cult., RBC 202 (UC): *rps4-trnS* JN189226*, psbA-trnH* JN189444, MMO03-313: *trnL*-*trnL-trnF* DQ514498*, rbcL* DQ508775.

***Phanerophlebia nobilis*** (Schltdl. & Cham.) C. Presl, Guerrero, Mexico, Rothfels 5 (DUKE): *rps4-trnS* JN189242*, psbA-trnH* JN189459, DQ514512*, rbcL* JN189569; Na, Yatskievych 17 (Na): *trnL-trnL-trnF* DQ514512; ***Phanerophlebia umbonata*** Underw., Na, Na, Yatskievych 16 (Na): *trnL-trnL-trnF* DQ514513; Nuevo León, Mexico, Yatskievych and Wollenweber 83-87 (IND): *rbcL* AF537233;.

***Polystichum attenuatum*** Tagawa & K. Iwats., Thailand, Middleton et al. 4942 (E): *rps4-trnS* KU244888*, psbA-trnH* KU244801, *trnL-trnL-trnF* KU244975*, rbcL* KU244725; ***Polystichum acanthophyllum*** (Franch.) Christ, Yunnan, China, Li Bing Zhang and Hai He 64 (MO): *rps4-trnS* KU244873*, psbA-trnH* KU244785*, trnL-trnL-trnF* KU244959*, rbcL* KU244712; ***Polystichum acutipinnulum*** Ching & K. H. Shing, Guangxi, China, Li Bing Zhang and Hai He 5401 (CDBI, CTC, MO): *rps4-trnS* KU244900, *trnL-trnL-trnF* KU244987*, rbcL* KU244732; ***Polystichum alcicorne*** (Baker) Diels, Chongqing, China, Hai He 919 (CTC): *rps4-trnS* KU244932*, psbA-trnH* KU244845*, trnL-trnL-trnF* KU245021*, rbcL* KU244759; Sichuan, China, Lu/EM33 (PYU): *rps4-trnS* DQ202466*,* *trnL-trnL-trnF* DQ202434*; ***Polystichum*** ***alcicorne*** (Baker) Diels, Guizhou, China, Li Bing Zhang 816 (CDBI, PYU): *rps4-trnS* KU244883*, psbA-trnH* KU244796*, trnL-trnL-trnF* KU244970*, rbcL* KU244723; ***Polystichum alfaroi*** (Christ) Barrington, Costa Rica, D. Barrington 1978 (VT): *trnL-trnL-trnF* EF177271**, rbcL* AF537236; ***Polystichum ammifolium*** (Poir.) C. Chr., Réunion, France, Le Péchon 1216 (CDBI): *rps4-trnS* KU244925*, psbA-trnH* KU244838*, trnL-trnL-trnF* KU245013*, rbcL* KU244752; Réunion, France, Le Péchon 1226 (CDBI): *rps4-trnS* KU244926*, psbA-trnH* KU244839*, trnL-trnL-trnF* KU245014*, rbcL* KU244753; Réunion, France, Le Péchon 1229 (CDBI): *rps4-trnS* KU244927*, psbA-trnH* KU244840, *trnL-trnL-trnF* KU245015*, rbcL* KU244754; ***Polystichum andersonii*** Hopkins, Washington state, USA, Sessa 39 (WIS): *rps4-trnS* JN189183*, psbA-trnH* JN189401, *trnL-trnL-trnF* JX476098**, rbcL* JN189510; ***Polystichum*** ***atkinsonii*** Bedd., Sichuan, China, Li Bing Zhang 4735 (CDBI, MO): *rps4-trnS* KU244860*, psbA-trnH* KU244770*, trnL-trnL-trnF* KU244945; ***Polystichum*** ***auriculum*** Ching, Yunnan, China, Li Bing Zhang and Hai He 5014 (CDBI, CTC), *rps4-trnS* KU244931*, psbA-trnH* KU244844, *trnL-trnL-trnF* KU245020*, rbcL* KU244758; ***Polystichum*** ***bakerianum*** (Atkins. ex C.B.Clarke) Diels, Sichuan, China, Hai He HG065 (CTC): *rps4-trnS* KU244893*, psbA-trnH* KU244806*, trnL-trnL-trnF* KU244980; ***Polystichum balansae*** Christ, Miyazaki, Japan TNS 771175 (TNS): *rbcL* AB575186; ***Polystichum biaristatum*** (Blume) T. Moore, Thailand, Middleton et al. 4739 (E): *rps4-trnS* KU244889*, psbA-trnH* KU244802*, trnL-trnL-trnF* KU244976; ***Polystichum*** ***bifidum*** Ching, Yunnan, China, Li Bing Zhang and Hai He 5012 (CDBI, CTC): *rps4-trnS* KU244930*, psbA-trnH* KU244843*, trnL-trnL-trnF* KU245019*, rbcL* KU244757; ***Polystichum*** ***bonseyi*** W. H. Wagner & Hobdy, Hawaii, USA, Driscoll 319 (Na): *trnL-trnL-trnF* EF177311**, rbcL* EF177341; ***Polystichum braunii*** (Spenn.) Fée, Hokkaido, Japan, TNS 765677 (TNS): *psbA-trnH* AB575806*, rbcL* AB575187; ***Polystichum californicum*** (D. C. Eaton) Diels, cult. at UC Berkeley Bot. Gard., 870059(18A) (UC): *rps4-trnS* KU244918*, psbA-trnH* KU244830, *trnL-trnL-trnF* KU245005, *rbcL* KU244745; ***Polystichum*** ***caruifolium*** (Baker) Diels, Sichuan, China, Li Bing Zhang and Cehong Li 4795 (CDBI, MO): *rps4-trnS* KU244884*, psbA-trnH* KU244797, *trnL-trnL-trnF* KU244971*, rbcL* KU244724; ***Polystichum castaneum*** (C. B. Clarke) B. K. Nayar & S. Kaur, Sichuan, China, Hai He HG091 (CTC): *rps4-trnS* KU244898*, psbA-trnH* KU244811*, trnL-trnL-trnF* KU244985*, rbcL* KU244730; ***Polystichum*** ***chingiae*** Ching, Yunnan, China, Li Bing Zhang and Hai He 4921 (CDBI, MO): *rps4-trnS* KU244880*, psbA-trnH* KU244793*, trnL-trnL-trnF* KU244967*, rbcL* KU244720; ***Polystichum*** ***christii*** Ching, SG Lu/H13, Na, China: *rps4-trnS* DQ151862*, trnL-trnL-trnF* DQ150399**, rbcL* AY545486; ***Polystichum*** ***chunii*** Ching, Guizhou, China, Li Bing Zhang and Hai He 586 (CDBI, CTC, MO): *rps4-trnS* KU244870*, psbA-trnH* KU244781*, trnL-trnL-trnF* KU244955; ***Polystichum concinnum*** Lellinger ex Barrington, Costa Rica, J. Kluge 1441 (VT): *rps4-trnS* KC890810, *trnL-trnL-trnF* EF177276**, rbcL* EF177320; ***Polystichum craspedosorum*** (Maxim.) Diels, Tokyo, Japan, TNS 764000 (TNS): *psbA-trnH* AB575808*, rbcL* AB575189; Zhangming Zhu 568, Henan, China (CDBI): *rps4-trnS* KU244935*, psbA-trnH* KU244848*, trnL-trnL-trnF* KU245024*, rbcL* KU244761; ***Polystichum*** ***crinigerum*** (C. Chr.) Ching, Yunnan, China, Li Bing Zhang and Hai He 4847 (CDBI, CTC, MO): *rps4-trnS* KU244882*, psbA-trnH* KU244795*, trnL-trnL-trnF* KU244969*, rbcL* KU244722; ***Polystichum*** ***cyclolobum*** C. Chr., Guizhou, China, Li Bing Zhang and Hai He 704 (CDBI, CTC, MO): *rps4-trnS* KU244857*, psbA-trnH* KU244767*, trnL-trnL-trnF* KU244942; ***Polystichum*** ***cystostegia*** (Hook.) J. B. Armstr., New Zealand, P20465 (WELT): *rps4-trnS* AY164630*, rbcL* AF208392; ***Polystichum deltodon*** (Baker) Diels, Sichuan, China, Li Bing Zhang et al. 4737 (CDBI, MO): *rps4-trnS* KU244863*, psbA-trnH* KU244773*, trnL-trnL-trnF* KU244948; Yunnan, China, Li Bing Zhang and Hai He 94 (CTC, MO): *rps4-trnS* KU244872*, psbA-trnH* KU244783*, trnL-trnL-trnF* KU244957*, rbcL* KU244710; Sichuan, China, Li Bing Zhang et al. 4749 (CDBI, MO): *rps4-trnS* KU244862*, psbA-trnH* KU244772*, trnL-trnL-trnF* KU244947*, rbcL* KU244703; Chongqing, China, He Hai Polystichum_nigrum_HG066 (CTC): *rps4-trnS* KU244890*, psbA-trnH* KU244803*, trnL-trnL-trnF* KU244977*, rbcL* KU244726; ***Polystichum dielsii*** Christ, Guizhou, China, Li Bing Zhang and Hai He 447 (CDBI, CTC, MO): *rps4-trnS* KU244871*, psbA-trnH* KU244782*, trnL-trnL-trnF* KU244956*, rbcL* KU244709; ***Polystichum*** ***discretum*** (D. Don) J. Smith, Yunnan, China, Li Bing Zhang and Hai He 4774 (CTC, MO): *psbA-trnH* KU244780*, trnL-trnL-trnF* KU244954; ***Polystichum*** ***dracomontanum*** Schelpe & N. C. Anthony, South Africa, Roux 2715 (NBG, VT), *trnL-trnL-trnF* EF177290**, rbcL* AF537240; ***Polystichum*** ***duthiei*** (C. Hope) C. Chr., Sichuan, China, Hai He HG087 (CTC): *rps4-trnS* KU244896*, psbA-trnH* KU244809*, trnL-trnL-trnF* KU244983; ***Polystichum ekmanii*** Maxon, Dominican Republic, P. Wieczorek 215 (VT): *rps4-trnS* KF020396, *trnL-trnL-trnF* EF177272**, rbcL* AF537242; ***Polystichum*** ***erosum*** Ching & K. H. Shing, Sichuan, China, Li Bing Zhang et al. 4775 (CDBI, MO): *rps4-trnS* KU244937*, psbA-trnH* KU244850*, trnL-trnL-trnF* KU245026*, rbcL* KU244763; ***Polystichum*** ***falcatilobum*** Ching ex W. M. Chu & Z. R. He, Sichuan, China, Li Bing Zhang et al. 4757 (CDBI, MO): *rps4-trnS* KU244861*, psbA-trnH* KU244771*, trnL-trnL-trnF* KU244946; ***Polystichum fallax*** Tindale, Australia, P20472 (WELT): *rps4-trnS* AY164625*, rbcL* AY163865; ***Polystichum* *fibrillosopaleaceum*** (Kodama) Tagawa, Shizuoka, Japan, TNS 769191 (TNS): *psbA-trnH* AB575811*, rbcL* AB575192; ***Polystichum*** ***fimbriatum*** Christ, Guizhou, China, Li Bing Zhang and Hai He 773 (CDBI, CTC, MO): *rps4-trnS* KU244859*, psbA-trnH* KU244769*, trnL-trnL-trnF* KU244944; ***Polystichum*** ***formosanum*** Rosenst., Taiwan, China, Ranker 2073 (COLO), *trnL-trnL-trnF* EF177307**, rbcL* EF177337; Japan, TNS 763920 (TNS): *psbA-trnH* AB575812*, rbcL* AB575194; ***Polystichum fraxinellum*** (Christ) Diels, Yunnan, China, LJM 002 (KUN): *trnL-trnL-trnF* AY736349*, rbcL* AY694810; ***Polystichum*** ***frigidicola*** H. S. Kung & Li Bing Zhang, Sichuan, China, Hai He HG071 (CTC): *rps4-trnS* KU244895*, psbA-trnH* KU244808*, trnL-trnL-trnF* KU244982*, rbcL* KU244729; ***Polystichum gracilipes*** C. Chr. var. ***gemmiferum*** Tagawa, Nagano, Japan, TNS 776367 (TNS): *psbA-trnH* AB575813*, rbcL* AB575195; ***Polystichum glaciale*** Christ., Taiwan, China, Fay-Wei Li 1395 (MO): *rps4-trnS* KU244917*, psbA-trnH* KU244829*, trnL-trnL-trnF* KU245004; ***Polystichum*** ***grandifrons*** C. Chr., Kagoshima, Japan, TNS 771415 (TNS): *psbA-trnH* AB575814*, rbcL* AB575196; Yunnan, China, Li Bing Zhang and Hai He 5025 (CDBI, CTC, MO): *rps4-trnS* KU244879*, psbA-trnH* KU244792*, trnL-trnL-trnF* KU244966*, rbcL* KU244719; ***Polystichum*** ***haleakalense*** Brack., Hawaii, USA, Driscoll 301 (VT), *trnL-trnL-trnF* EF177278**, rbcL* EF177322; ***Polystichum hancockii*** (Hance) Diels, Okinawa, Japan, TNS 763908 (TNS): *psbA-trnH* AB575815*, rbcL* AB575197; ***Polystichum hecatopterum*** Diels, Sichuan, China, Liang Zhang s.n. (CDBI): *rps4-trnS* KU244939*, psbA-trnH* KU244853*, trnL-trnL-trnF* KU245030; Sichuan, China, Li Bing Zhang 5717 (CDBI, MO): *psbA-trnH* X; ***Polystichum herbaceum*** Ching & Z. Y. Liu, Chongqing, China, Liang Zhang and Zhangming Zhu 1012 (CDBI): *rps4-trnS* KU244906*, psbA-trnH* KU244818*, trnL-trnL-trnF* KU244993; ***Polystichum hillebrandii*** Carruth., Hawaii, USA, Driscoll 315 (VT): *trnL-trnL-trnF* EF177279**,* Driscoll 310: *rbcL* EF463217; ***Polystichum*** ***hookerianum*** (Pr.) C. Chr., Kagoshima, Japan, TNS 771431 (TNS): *psbA-trnH* AB575816*, rbcL* AB575198; Guangxi, China, Li Bing Zhang and Hai He 5394 (CDBI, CTC, MO): *rps4-trnS* KU244908*, psbA-trnH* KU244820*, trnL-trnL-trnF* KU244995*, rbcL* KU244738; ***Polystichum hubeiense*** Liang Zhang & Li Bing Zhang, Hubei, China, Liang Zhang and Zhangming Zhu 1044 (CDBI, MO): *psbA-trnH* KU244852*, trnL-trnL-trnF* KU245029; Hubei, China, X.C. Zhang 3341: *rps4-trnS* EF540715, *trnL-trnL-trnF* EF540703; ***Polystichum*** ***igaense*** Tagawa, Tokyo, Japan, TNS 763928 (TNS): *psbA-trnH* AB575817*, rbcL* AB575199; ***Polystichum imbricans*** (D. C. Eaton) D. H. Wagner, cult. at UC Berkeley Bot. Gard., acc. #20110803 (UC): *rps4-trnS* KU244919*, psbA-trnH* KU244831, *trnL-trnL-trnF* KU245006*, rbcL* KU244746; ***Polystichum incongruum*** J. P. Roux, South Africa, Roux 5414 (NBG): *rps4-trnS* KU244921*, psbA-trnH* KU244834*, trnL-trnL-trnF* KU245009*, rbcL* KU244748; South Africa, Roux 5448 (NBG): *rps4-trnS* KU244922*, psbA-trnH* KU244835*, trnL-trnL-trnF* KU245010*, rbcL* KU244749; ***Polystichum jiucaipingense*** P.S. Wang & Q. Luo, Guizhou, China, Li Bing Zhang and Hai He 700 (CDBI, CTC, MO): *rps4-trnS* KU244856*, psbA-trnH* KU244766*, trnL-trnL-trnF* KU244941*, rbcL* KU244702; ***Polystichum jiulaodongense*** W. M. Chu & Z. R. He, Sichuan, China, Li Bing Zhang 4771 (CDBI, MO): *rps4-trnS* KU244938*, psbA-trnH* KU244851*, trnL-trnL-trnF* KU245027; ***Polystichum kungianum*** H. He & Li Bing Zhang, Chongqing, China, Hai He and Yongqing Yang 016 (CTC): *trnL-trnL-trnF* KU245028*, rbcL* KU244764; ***Polystichum*** ***lachenense*** (Hook.) Bedd., Taiwan, China, Fay-Wei Li 1403 (MO): *rps4-trnS* KU244915*, psbA-trnH* KU244827*, trnL-trnL-trnF* KU245002*, rbcL* KU244743; Sichuan, China, Hai He HG090 (CTC): *rps4-trnS* KU244897*, psbA-trnH* KU244810*, trnL-trnL-trnF* KU244984; Nagano, Japan, TNS 766487 (TNS): *psbA-trnH* AB575819*, rbcL* AB575201; ***Polystichum*** ***latilepis*** Ching & H. S. Kung, Hubei, China, Liang Zhang and Zhangming Zhu 1083 (CDBI): *rps4-trnS* KU244904*, psbA-trnH* KU244816*, trnL-trnL-trnF* KU244991*, rbcL* KU244735; ***Polystichum*** ***lentum*** (D. Don) T. Moore, Himalaya, P20477 (WELT): *rps4-trnS* AY164637; Cult. NYBG 474/77 (VT): *trnL-trnL-trnF* EF177293**, rbcL* AF537246; ***Polystichum lepidocaulon*** (Hook.) Bedd. Tokyo, Japan, TNS 764341 (TNS): *psbA-trnH* AB575820, *rbcL* AB575202; Yunnan China, SG Lu/Q12 (PYU):*rps4-trnS*DQ151855, *trnL-trnL-trnF* DQ150392*; Na, China, LJM181 (KUN): *rbcL* DQ508767; Cult. NYBG 570/76 (VT): *trnL-trnL-trnF* EF177266*, *rbcL* AF537224; ***Polystichum lepidotum*** M. Sundue & M. Kessler; M. A. McHenry 10-94 (VT): *rps4-trnS* KF020415, *trnL-trnL-trnF* KF020367*, *rbcL* KF020338; ***Polystichum*** ***lonchitis*** (L.) Roth, France, TNS 743763 (TNS): *rbcL* AB575203, AEG; Alaska, USA, Little 344 (VT): *rbcL* AF537247, Washington, USA, Zika18981 (VT): *trnL-trnL-trnF* AY736354, AEG; ***Polystichum*** ***longifrons*** Sa. Kurata, Ibaraki, Japan, TNS 774851 (TNS): *psbA-trnH* AB575821*, rbcL* AB575204; ***Polystichum*** ***longispinosum*** Ching ex Li Bing Zhang & H.S. Kung, Sichuan, China, Li Bing Zhang and Cehong Li 4786 (CDBI, MO): *rps4-trnS* KU244867*, psbA-trnH* KU244777*, trnL-trnL-trnF* KU244951*, rbcL* KU244706; ***Polystichum*** ***luctuosum*** (Kunze) T. Moore, South Africa, Roux 5494 (VT): *rps4-trnS* KU244923*, psbA-trnH* KU244836*, trnL-trnL-trnF* KU245011*, rbcL* KU244750; ***Polystichum macleaii*** (Baker) Diels, South Africa, Roux 2561 (VT): *trnL-trnL-trnF* EF177294**, rbcL* AF537249; ***Polystichum******maevaranense*** Tardieu, Mahajanga, Madagascar, Rakotovao et al. 2344 (MO, P, TAN): *psbA-trnH* KU244833*, trnL-trnL-trnF* KU245008*, rbcL* KU244747; ***Polystichum*** ***makinoi*** (Tagawa) Tagawa, Kanagawa, Japan, TNS 764342 (TNS): *psbA-trnH* AB575822*, rbcL* AB575205; Yunnan, China, Lu/C61 (PYU): *rps4-trnS* DQ202462, *trnL-trnL-trnF* DQ202431**, rbcL* AY545494; ***Polystichum manmiense*** (Christ) Nakaike, Yunnan, China, Li Bing Zhang and Hai He 4911 (CDBI, CTC, MO): *rps4-trnS* KU244877*, psbA-trnH* KU244790*, trnL-trnL-trnF* KU244964*, rbcL* KU244717; ***Polystichum mayebarae*** Tagawa, Tokyo, Japan, TNS 765125 (TNS): *psbA-trnH* AB575839*, rbcL* AB575224; ***Polystichum maximum*** M. Kessler & A. R. Sm.; M. A. McHenry 10-84 (VT): *rps4-trnS* KF020416, *trnL-trnL-trnF* KF020368*, *rbcL* KF020318; ***Polystichum*** ***microchlamys*** (Christ) Kodama var. ***azumiense*** Seriz, Akita, Japan, TNS 765798 (TNS), *psbA-trnH* AB575207, *rbcL* AB575207; ***Polystichum minimum*** (Y. T. Hsieh) Li Bing Zhang, Chongqing, China, LJM 047 (KUN): *trnL-trnL-trnF* AY736351*, *rbcL* AY694812; ***Polystichum*** ***munitum*** (Kaulf.) C. Presl, Washington state, USA, Sessa 34 (WIS): *rps4-trnS* JN189182*, psbA-trnH* JN189399, *trnL-trnL-trnF* JN105309**, rbcL* JN189508; cult., Pud 21B 20050024 (UC): *rps4-trnS* KU244920*, psbA-trnH* KU244832, *trnL-trnL-trnF* KU245007; ***Polystichum*** ***mohrioides*** (Bory ex Willd.) C. Presl, Chile, Connolly 2 (VT): *trnL-trnL-trnF* EF177314**, rbcL* AF537250; ***Polystichum*** ***montevidense*** (Spreng.) Rosenst., Bolivia, Sundue 621 (VT): *trnL-trnL-trnF* EF177282**, rbcL* EF177326; ***Polystichum*** ***moorei*** Christ, Lord Howe Island, P20475 (WELT): *rps4-trnS* AY164628, *trnL-trnL-trnF* KU245033; ***Polystichum*** ***moupinense*** (Franch.) Bedd., Sichuan, China, Hai He HG118 (CTC): *rps4-trnS* KU244899*, psbA-trnH* KU244812*, trnL-trnL-trnF* KU244986, *rbcL* KU244731; ***Polystichum*** ***mucronifolium*** (Blume) C. Presl, Yunnan, China, Li Bing Zhang and Hai He 4922 (CDBI, CTC, MO): *rps4-trnS* KU244887*, psbA-trnH* KU244800*, trnL-trnL-trnF* KU244974; Yunnan, China, Li Bing Zhang and Hai He 4912 (CDBI, CTC, MO): *rps4-trnS* KU244875*, psbA-trnH* KU244788*, trnL-trnL-trnF* KU244962*, rbcL* KU244715; ***Polystichum*** ***muricatum*** (L.) Fée, Costa Rica, Little 349 (VT): *rps4-trnS* KF020404, *trnL-trnL-trnF* EF177275**, rbcL* AF537251; ***Polystichum*** ***neolobatum*** Nakai, Nagano, Japan, TNS 743679 (TNS)*: rbcL* AB575208; ***Polystichum*** ***nepalense*** (Spr.) C. Chr., Yunnan, China, Li Bing Zhang and Hai He 4928: *psbA-trnH* KU244786*, trnL-trnL-trnF* KU244960*, rbcL* KU244713; ***Polystichum*** ***nigrum*** Ching & H. S. Kung, Sichuan, China, Hai He HG066 (CTC): *rps4-trnS* KU244894*, psbA-trnH* KU244807*, trnL-trnL-trnF* KU244981*, rbcL* KU244728; ***Polystichum*** ***obae*** Tagawa, Kagoshima, Japan, TNS 766461 (TNS): *psbA-trnH* AB575824*, rbcL* AB575209; ***Polystichum*** ***oculatum*** (Hook.) J. B. Armstr., New Zealand, P20327 (WELT): *rps4-trnS* AY164633 *trnL-trnL-trnF* JX476120*; ***Polystichum*** ***otomasui*** Kurata,, Miyazaki, Japan, TNS 762702 (TNS): *psbA-trnH* AB575826*, rbcL* AB575211; ***Polystichum*** ***otophorum*** (Franch.) Bedd., Sichuan, China, Li Bing Zhang and Hai He 6 (CTC, MO): *psbA-trnH* KU244784*, trnL-trnL-trnF* KU244958*, rbcL* KU244711; ***Polystichum*** ***ovatopaleaceum*** (Kodama) Sa. Kurata, Shizuoka, Japan, TNS 9508214 (TNS): *psbA-trnH* AB575827*, rbcL* AB575212; ***Polystichum*** ***parvipinnulum*** Tagawa, Taiwan, China, s.c. 22959 (TNS): *rps4-trnS* KU244910*, psbA-trnH* KU244822*, trnL-trnL-trnF* KU244997*, rbcL* KU244739; Knapp 3516 (herb. R. Knapp): *trnL-trnL-trnF* KU245018; ***Polystichum*** ***piceopaleaceum*** Tagawa, Nepal, TNS 763912 (TNS): *psbA-trnH* AB575829*, rbcL* AB575214; ***Polystichum*** ***platyphyllum*** (Willd.) C. Presl, Costa Rica, Barrington 2099 (VT): *rps4-trnS* KF02042, *trnL-trnL-trnF* EF177285**, rbcL* EF177329; ***Polystichum*** ***polyblepharum*** (Roemer ex Kunze) C. Presl, Kagoshima, Japan, TNS 762608 (TNS): *psbA-trnH* AB575830*, rbcL* AB575215; ***Polystichum*** ***polyblepharum*** (Roemer ex Kunze) C. Presl, cult. at Benmore Fernery, Li Bing Zhang 5298 (MO): *rps4-trnS* KU244885*, psbA-trnH* KU244798*, trnL-trnL-trnF* KU244972; ***Polystichum polyphyllum*** C. Presl, South America, M. A. McHenry 10-51 (VT): *rps4-trnS* KF020418, *trnL-trnL-trnF* KF020374*, *rbcL* KF020342; ***Polystichum*** ***proliferum*** (R. Br.) C. Presl, Australia, WELT P20471 (WELT): *rps4-trnS* AY164627, *rbcL* AF208393: *trnL-trnL-trnF* KU245031; ***Polystichum*** ***pseudomakinoi*** Tagawa, Na, Japan, TNS 763861 (TNS): *psbA-trnH* AB575832*, rbcL* AB575217; ***Polystichum*** ***pseudoxiphophyllum*** Ching ex H. S. Kung, Guizhou, China, Li Bing Zhang and Hai He 707 (CDBI, CTC, MO): *rps4-trnS* KU244858*, psbA-trnH* KU244768*, trnL-trnL-trnF* KU244943; ***Polystichum*** ***putuoense*** Li Bing Zhang, Na, China, H. Zhang BY03 (Na): *rps4-trnS* EF540710*, trnL-trnL-trnF* EF540697, *rbcL* EF463124; ***Polystichum*** ***pycnopterum*** (Christ) Ching ex W. M. Chu & Z. R. He, Yunnan, China, Li Bing Zhang and Hai He 4938 (CDBI, CTC, MO): *rps4-trnS* KU244874*, psbA-trnH* KU244787*, trnL-trnL-trnF* KU244961*, rbcL* KU244714; Yunnan, China, Lu/B11 (PYU): *rps4-trnS* DQ151876, *trnL-trnL-trnF* DQ150414**, rbcL* AY545502; ***Polystichum*** ***retrosopaleaceum*** (Kodama) Tagawa, Kyoto, Japan, TNS 9508176 (TNS): *psbA-trnH* AB575833*, rbcL* AB575218; ***Polystichum*** ***revolutum*** P. S. Wang, Sichuan, China, Li Bing Zhang and Cehong Li 4773 (CDBI, CTC, MO): *rps4-trnS* KU244869*, psbA-trnH* KU244779*, trnL-trnL-trnF* KU244953*, rbcL* KU244708; ***Polystichum*** ***rigens*** Tagawa, Hubei, China, Liang Zhang and Zhangming Zhu 1082 (CDBI): *rps4-trnS* KU244905*, psbA-trnH* KU244817*, trnL-trnL-trnF* KU244992*, rbcL* KU244736; Tokyo, Japan, TNS 764383 (TNS): *psbA-trnH* AB575834*, rbcL* AB575219; ***Polystichum scariosum*** (Roxb.) C. V. Morton, Kagoshima, Japan, TNS 764380 (TNS): *psbA-trnH* AB575810*, rbcL* AB575191; ***Polystichum*** ***semifertile*** (Clarke) Ching, Yunnan, China, Li Bing Zhang and Hai He 4903 (CDBI, CTC, MO): *rps4-trnS* KU244878*, psbA-trnH* KU244791*, trnL-trnL-trnF* KU244965*, rbcL* KU244718; ***Polystichum*** ***setiferum*** (Forssk.) Moore ex Woynar, Europe, P20478 (WELT): *rps4-trnS* AY164638, EB700: *trnL-trnL-trnF* HQ676535; Europe, cult. NYBG (VT): *rbcL* AF537254; cult. at Benmore Fernery, Li Bing Zhang 5296 (MO): *rps4-trnS* KU244886*, psbA-trnH* KU244799*, trnL-trnL-trnF* KU244973; ***Polystichum*** ***sp.*** Christ, Sichuan, China, Li Bing Zhang and Hai He 39 (CTC, MO): *rps4-trnS* KU244866*, psbA-trnH* KU244776*, trnL-trnL-trnF* KU244950; ***Polystichum*** cf. ***sinense*** (Christ) Christ, Sichuan, China, Hai He HG004 (CTC): *rps4-trnS* KU244891*, psbA-trnH* KU244804*, trnL-trnL-trnF* KU244978*, rbcL* KU244727; ***Polystichum*** ***sinense*** (Christ) Christ, Réunion, France, Le Péchon 1225 (CDBI, REU): *rps4-trnS* KU244928*, psbA-trnH* KU244841, *trnL-trnL-trnF* KU245016*, rbcL* KU244755; Réunion, France, Le Péchon 1227 (CDBI, REU): *rps4-trnS* KU244929*, psbA-trnH* KU244842, *trnL-trnL-trnF* KU245017*, rbcL* KU244756; South Africa, Roux 5535 (NBG): *rps4-trnS* KU244924*, psbA-trnH* KU244837, *trnL-trnL-trnL* KU245012*, rbcL* KU244751; ***Polystichum*** ***sinotsus-simense*** Ching & Z. Y. Liu, Guangxi, China, Li Bing Zhang and Hai He 5690 (CDBI, CTC, MO): *rps4-trnS* KU244901*, psbA-trnH* KU244813, *trnL-trnL-trnF* KU244988*, rbcL* KU244733; ***Polystichum*** ***sozanense*** Ching ex H. S. Kung & Li Bing Zhang, Taiwan, China, Pi-Fong Lu 23670 (TFRI): *rps4-trnS* X*, psbA-trnH* X*, trnL-trnL-trnF:* X; ***Polystichum*** ***speciosissimum*** (A. Braun ex Kunze) R. M. Tryon & A. F. Tryon, Costa Rica, Little 297 (VT): *trnL-trnL-trnL* DQ514517*,* *rps4-trnS* KF020397; Mexico, Barrington 2027 (VT): *rbcL* AF537255; ***Polystichum*** ***stenophyllum*** (Franch.) Christ, Sichuan, China, Li Bing Zhang 4727 (CDBI, MO): *rps4-trnS* KU244864*, psbA-trnH* KU244774, *rbcL* KU244704; Sichuan, China, Hai He HG006 (CTC): *rps4-trnS* KU244892*, psbA-trnH* KU244805, *trnL-trnL-trnF* KU244979; ***Polystichum*** ***subfimbriatum*** W. M. Chu & Z. R. He, Yunnan, China, Li Bing Zhang and Hai He 4837 (CDBI, CTC, MO): *rps4-trnS* KU244881*, psbA-trnH* KU244794, *trnL-trnL-trnF* KU244968*, rbcL* KU244721; ***Polystichum*** ***submarginale*** (Baker) Ching ex P. S. Wang, Sichuan, China, Li Bing Zhang and Cehong Li 4793 (CDBI, MO): *rps4-trnS* KU244868*, psbA-trnH* KU244778*, trnL-trnL-trnF* KU244952*, rbcL* KU244707; ***Polystichum*** ***tagawanum*** Sa. Kurata, Shizuoka, Japan, TNS 9508172 (TNS): *psbA-trnH* AB575836*, rbcL* AB575221; ***Polystichum*** ***talamancanum*** Barrington, Costa Rica, Little and Barrington 299 (VT): *rps4-trnS* KF020402, *trnL-trnL-trnF* EF177305**, rbcL* EF177335; ***Polystichum*** ***tenuius*** (Ching) Li Bing Zhang, Chongqing, China, Liang Zhang 160 (CDBI): *rps4-trnS* KU244865*, psbA-trnH* KU244775, *trnL-trnL-trnL* KU244949*, rbcL* KU244705; Guangxi, China, Li Bing Zhang and Hai He 5691 (CDBI, CTC, MO): *rps4-trnS* KU244902*, psbA-trnH* KU244814, *trnL-trnL-trnF* KU244989; Guizhou, China, Li-Bing Zhang and Hai He 6206 (CDBI): *rps4-trnS* KU244934*, psbA-trnH* KU244846, *trnL-trnL-trnL* KU245022; Yunnan, China, Lu SG J29 (PYU): *rps4-trnS* DQ202440, *trnL-trnL-trnL* DQ202409*; ***Polystichum*** ***thomsonii*** (J.D. Hook.) Bedd., Taiwan, China, Fay-Wei Li 1391 (MO): *rps4-trnS* KU244916*, psbA-trnH* KU244828, *trnL-trnL-trnF* KU245003*, rbcL* KU244744; Yunnan, China, Li Bing Zhang and Hai He 4824 (CDBI, CTC, MO): *rps4-trnS* KU244876*, psbA-trnH* KU244789, *trnL-trnL-trnF* KU244963*, rbcL* KU244716; ***Polystichum*** ***tonkinense*** (Christ) W. M. Chu & Z. R. He, Guizhou, China, Li-Bing Zhang et al. 473 (CDBI, CTC, MO): *rps4-trnS* KU244933*, psbA-trnH* KU244847, *trnL-trnL-trnF* KU245023*, rbcL* KU244760; ***Polystichum*** ***transkeiense*** W. Jacobsen, South Africa, Roux 2493 (VT): *trnL-trnL-trnF* EF177297**, rbcL* AF537257; ***Polystichum*** ***tripteron*** (Kunze) C. Presl, Kagoshima, Japan, TNS 763162 (TNS): *psbA-trnH* AB575837*, rbcL* AB575222; ***Polystichum*** ***tsus-simense*** (Hook.) J. Smith, Sichuan, China, Hai He 1024 (CTC): *rps4-trnS* KU244903*, psbA-trnH* KU244815, *trnL-trnL-trnF* KU244990*, rbcL* KU244734; Kumamoto, Japan, TNS 762664 (TNS): *psbA-trnH* AB575838*, rbcL* AB575223; ***Polystichum*** ***vestitum*** (G. Forst.) C. Presl, New Zealand, P20468 (WELT): *rps4-trnS* AY164635, *rbcL* AF208395; New Zealand, Vogel s.n. (BM): *trnL-trnL-trnF* AY300046*; ***Polystichum*** ***wawranum*** (Szyszył.) Perrie, New Zealand, P20308 (WELT): *rps4-trnS* AY164636, New Zealand P020311 (WELT): *trnL-trnL-trnF*: KU245032; ***Polystichum*** ***weimingii*** Li Bing Zhang & H. He, Yunnan, China, Li Bing Zhang et al. 5985 (CDBI, CTC, MO): *rps4-trnS* KU244909*, psbA-trnH* KU244821, *trnL-trnL-trnF* KU244996*, rbcL* KU253818; ***Polystichum*** ***xichouense*** (S. K. Wu & Mitsuta) Li Bing Zhang, Yunnan, China, SG Lu/J30 (PYU): *rps4-trnS* DQ202441*,* *trnL-trnL-trnF* EU106595**, rbcL* DQ054515; ***Polystichum*** ***xiphophyllum*** (Baker) Diels, Guizhou, China, Li Bing Zhang et al. 636 (CDBI, CTC, MO): *rps4-trnS* KU244855*, psbA-trnH* KU244765, *trnL-trnL-trnF* KU244940*, rbcL* KU244701; ***Polystichum* *yaanense***Liang Zhang & Li Bing Zhang, Sichuan, China, Li Bing Zhang et al. 4745 (CDBI, MO): *rps4-trnS* KU244936*, psbA-trnH* KU244849, *trnL-trnL-trnF* KU245025*, rbcL* KU244762; ***Polystichum*** ***yaeyamense*** Makino, Okinawa, Japan, TNS 759286 (TNS): *psbA-trnH* AB575840*, rbcL* AB575225; ***Polystichum yuanum*** Ching, Yunnan, China, W. M. Zhu and Z. R. He *s.n.* (CDBI, PYU): *rps4-trnS* KU244907*, psbA-trnH* KU244819, *trnL-trnL-trnF* KU244994*, rbcL* KU244737.
